# Geochemical, radiological, and heat-production characteristics of the ElGara granitoids (Southwestern Desert)

**DOI:** 10.1038/s41598-026-35954-z

**Published:** 2026-02-09

**Authors:** Ghada Salaheldin, Mostafa K. Seddeek, Fuad Ameen, Chithra Sivanandan, Mervat A. Elhaddad

**Affiliations:** 1 Department of Physics, Faculty of Science, Assiut University, Assiut, 71516 Egypt; 2 Department of Physics, Faculty of Science, Arish University, Arish, 45511 Egypt; 3 Department of Botany and Microbiology, College of Science, King Saud University, 11451 Riyadh, Saudi Arabia; 4 Department of Biotechnology of Medicines, Warsaw University of Technology, Warsaw, Poland; 5 Department of Geology, Faculty of Science, Assiut University, Assiut, 71516 Egypt

**Keywords:** A-type granitoids, Radiogenic heat production, Geochemistry, Radiological assessment, Peraluminous, Peralkaline, Arabian–Nubian shield, Gamma spectrometry, Environmental sciences, Solid Earth sciences

## Abstract

This study provides an integrated geochemical, petrographic, and radiological assessment of the El Gara El Hamra and El Gara El Soda granitoids in Egypt’s Southwestern Desert. Whole-rock major, trace, and REE geochemistry, combined with tectonic discrimination diagrams, reveals that the granitoids belong to ferroan A-type suites and comprise both peraluminous and peralkaline varieties. These contrasting chemistries reflect heterogenous crustal sources and within-plate magmatic processes associated with late Neoproterozoic post-collisional extension. Elemental ratios (e.g., Nb/Yb, Ga/Al) and HFSE enrichments support an anhydrous, oxidized, high-temperature melt regime consistent with the regional evolution of the Arabian–Nubian Shield. High-resolution gamma spectrometry was used to quantify primordial radionuclides (^238^U, ^232^Th, ^40^K). Thorium and potassium show pronounced enrichment in the peralkaline samples, whereas uranium displays moderate variability across the granitoid suites. Calculated radiological parameters—including absorbed dose rate (D_γ_), annual effective dose (E_annual), radium equivalent activity (Ra_eq_), and hazard indices—exceed global crustal averages but remain within ranges typical of A-type granites worldwide. Radiogenic heat production (RHP) varies significantly between the peraluminous and peralkaline groups, reaching up to 9.99 µW/m^3^, indicating favorable potential for shallow-crust geothermal exploration. Organ-specific dose modeling (ICRP-based) identifies the bone marrow and lungs as the most impacted tissues under hypothetical prolonged exposure scenarios. Although some samples exceed recommended limits for unrestricted building use, actual public exposure would depend on rock utilization and exposure geometry rather than intrinsic radionuclide concentrations alone. Overall, the El Gara granitoids represent a compositionally diverse A-type system with elevated heat-producing elements and moderate radiological significance. These findings highlight the need for site-specific radiological evaluation before large-scale quarrying or use as construction materials, and underscore their potential relevance for geothermal energy assessments.

## Introduction

Natural background radiation arises predominantly from the long-lived primordial radionuclides ^238^U, ^232^Th, and ^40^K, which together account for nearly 80% of the dose received by humans^1^. These radionuclides occur naturally in soils, aquifers, and crustal rocks, and their concentrations vary according to lithology, mineralogy, and weathering history^2,3^. Unlike these parents, ^226^Ra is not a primordial radionuclide; it is a short-lived intermediate daughter in the ^238^U decay chain (t^1/2^ ≈ 1600 year), but it is routinely measured by gamma spectrometry because it serves as a practical proxy for ^238^U under secular-equilibrium conditions. Thorium occurs almost entirely as ^232^Th (t^1/2^ = 1.41 × 10^10^ yr), and the trace isotope ^40^K (~ 0.012% of natural K) contributes significantly to terrestrial gamma radiation^4^. Concentrations of these heat-producing elements (HPEs) are typically highest in felsic magmatic systems enriched in zircon, allanite, monazite, and other accessory minerals that host U, Th, and K^5^.

Granitoids are, therefore, of primary interest in natural radioactivity studies, as numerous investigations have shown elevated concentrations of radioelements in granites of the Egyptian Shield, including Gattar, Missikat, Abu Dabbab, and Nuweibi^6–11^. These plutons often exhibit enhanced radiogenic heat production (RHP), influencing crustal thermal gradients, metamorphic conditions, and geothermal potential^12–15^. Global datasets show systematic variation in HPE content depending on granite type, source composition, emplacement age, and degree of magmatic differentiation, with younger and more evolved granites commonly exhibiting higher U–Th–K concentrations^16,17^.

Previous gamma-spectrometric studies across the Eastern Desert, Egypt have documented large variability in radioelement concentrations and radiological hazard indices among different granite suites, reflecting differences in mineralogy, petrogenesis, and potential mineralization^7,18–21^. These findings provide essential context for evaluating both environmental hazards and geothermal potential in lesser-studied regions such as the Southwestern Desert, where baseline radiological data remain sparse^22–24^.

From a thermal perspective, spatial heterogeneities in RHP within granitic bodies can generate local heat-flow anomalies relevant to geothermal exploration. Consequently, RHP measurements are now routinely integrated into subsurface thermal modeling and regional heat-flow assessments^23,25^. Similarly, radiological indices such as radium equivalent activity, gamma dose rate, annual effective dose, and external hazard indices allow comparison with international safety guidelines (UNSCEAR, ICRP) and help determine the suitability of rocks for construction or human exposure^26,27^.

A growing interdisciplinary theme involves the potential role of radioresistant microorganisms—especially *Deinococcus radiodurans*—in radionuclide immobilization and biological shielding. While no biological experiments were conducted on the El Gara granitoids, the broader literature suggests possible future applications of extremophiles for bioremediation and for reducing radionuclide mobility in contaminated or high-radiation environments^28–33^. In the present study, this concept is discussed solely as a theoretical extension, not as an experimental component.

Despite their geological significance, the El Gara ElHamra and El Gara ElSoda plutons in the Southwestern Desert have never been comprehensively evaluated in terms of their combined geochemical affinity, radiogenic heat production, and radiological risk profile. This gap is notable because the granitoids exhibit A-type characteristics and may represent important crustal heat-producing bodies analogous to other high-RHP granites in Egypt.

Therefore, the present study addresses a clear research gap, as no previous work has simultaneously integrated detailed geochemical classification, petrogenetic interpretation, quantified radiogenic heat production, and full radiological hazard assessment for the El Gara granitoids. Accordingly, the objectives of this study are to:

1. Classify the El Gara granitoids and constrain their A-type affinity and petrogenetic origins.
2. Quantify activity concentrations of ^232^Th, ^40^K, and ^226^Ra (as the measured gamma-spectrometric proxy for the ^238^U series).
3. Calculate radiogenic heat production (RHP) and evaluate its spatial variability.
4. Assess radiological hazard indices and environmental risk implications.
5. Provide a theoretical discussion of biological radiation mitigation based on radioresistant microorganisms, consistent with the literature but not presented as an applied or experimental component.

## Geological setting

The ElGara ElHamra (Gh) and ElGara ElSoda (Gs) (23°24′–23°52′N, 31°22′–31°50′E) stocks are situated in the Southwestern Desert of Egypt, forming part of the northern Arabian–Nubian Shield (ANS). The region exposes a Neoproterozoic basement complex composed of metavolcanic and metasedimentary assemblages intruded by several generations of granites^34–36^.

### Regional and tectonic framework of elgara granitoids

The Arabian–Nubian Shield in Egypt records a complex magmatic and tectonic history spanning 870–550 Ma, marking the assembly and stabilization of the East African Orogen. The studied granitoids represent post-tectonic intrusions emplaced during the waning stages of the Pan-African event (~ 580–600 Ma), contemporaneous with other A-type granites across the ANS^37–39^. These intrusions preceded the breakup of Gondwana and the later opening of the Red Sea (~ 50 Ma)^39,40^. El Gara granitoids were emplaced during the late- to post-orogenic transition (ca. 600–580 Ma), a period characterized by a shift from compressional to extensional tectonic regimes^41,42^. This stage witnessed widespread emplacement of A-type granites derived from partial melting of crustal sources with minor mantle contribution, commonly associated with transtensional shear zones and uplift^42,43^. The basement complex evolved through successive arc accretion, crustal amalgamation, and late-stage post-orogenic extension during the Neoproterozoic Pan-African Orogeny (~ 870–550 Ma)^41,43^.

### Local geology and field relations

**Fig. 1:**
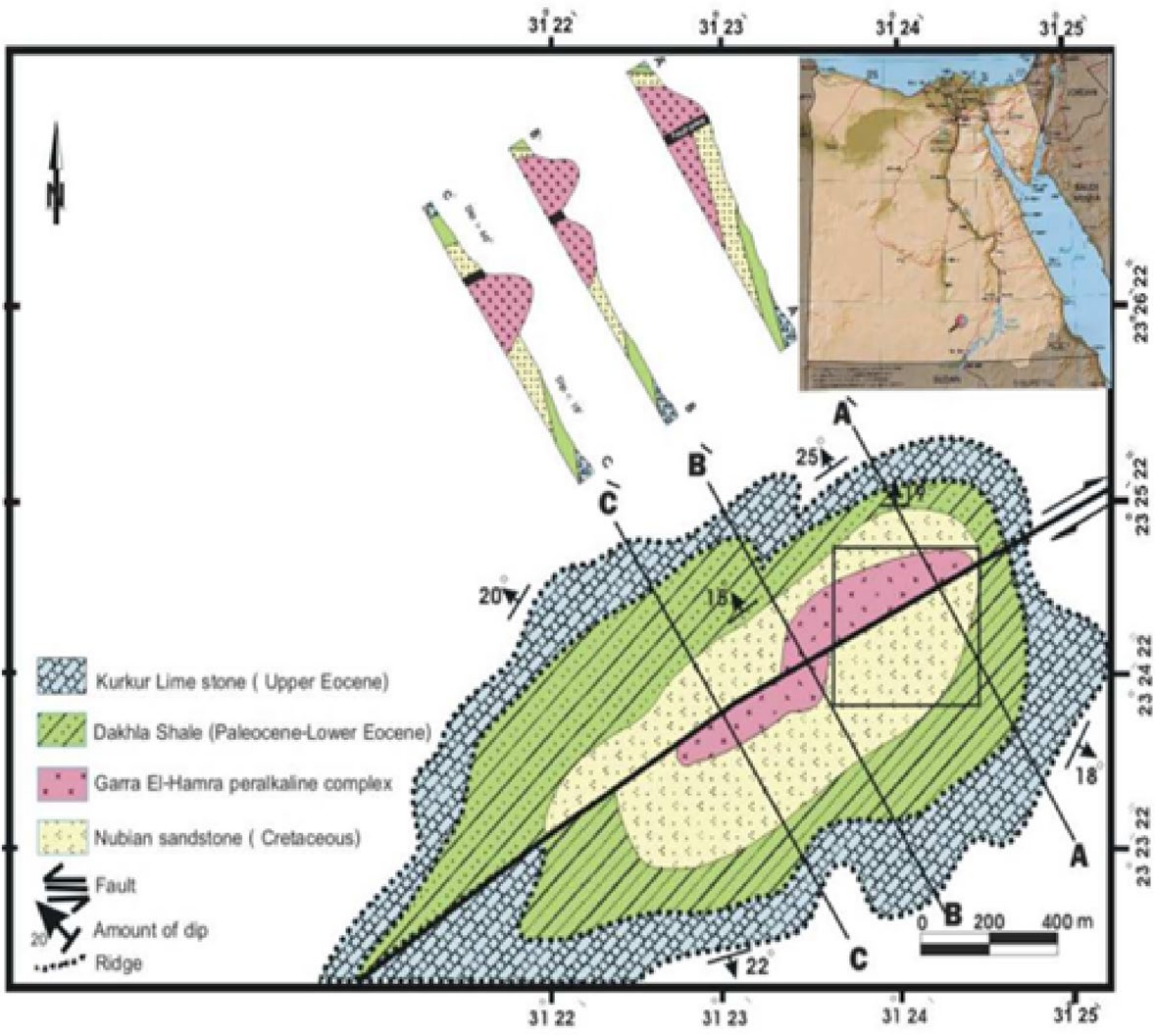
Simplified geological sketch map of the El Gara El Hamra intrusion, showing the main lithological units and three cross sections (A–A′, B–B′, and C–C′) oriented normal to the major NE–SW fault zone^44^.

The El Gara El Hamra intrusion is an isolated, oval-shaped syenitic hill (~ 0.3 km^2^) located ~ 170 km southwest of Aswan, near the Kurkur and Dungul oases. It is bordered by Cretaceous–Eocene sedimentary rocks. Three main lithological units are observed (Fig. 1): An early volcanic pile of alkaline trachyte and rhyolite porphyries; Intrusions of peralkaline syenite with subordinate granitic apophyses; Peripheral zones containing xenoliths of Nubian Sandstone.

The intrusion follows a NE–SW-trending fault system, further dissected by minor N–S fractures infilled with quartz, barite, and fluorite veins. Alteration features such as hematitization, silicification, and hydrothermal mineralization are common, indicating post-emplacement fluid activity. Approximately 4 km southwest, the El Gara El Soda stock lies along the same structural trend and covers ~ 3.15 km^2^. It consists mainly of syenite, locally intruded by basic dykes and minor gabbroic lenses in its northern part. The intrusion postdates the Nubian Sandstone, confirming post-Cretaceous uplift and exposure, although its magmatic crystallization age corresponds to the Neoproterozoic basement^24^. Field relationships suggest emplacement controlled by regional strike-slip fault systems, followed by cross-cutting quartz diorite and jasperoid veins.

### Petrography and magmatic evolution

The ElGara granitoids are medium- to coarse-grained, ranging in composition from syenite to quartz monzonite. They exhibit sharp intrusive contacts with surrounding rocks and lack ductile deformation, supporting a post-orogenic emplacement environment. The rocks display massive to weakly foliated textures and well-developed joint sets trending NW–SE and NE–SW. Subvertical cooling joints and occasional radial jointing near pluton centers reflect late-stage solidification processes. Mineralogically, the granitoids consist mainly of quartz, alkali feldspar, and plagioclase, with accessory zircon, apatite, titanite, and allanite. Minor biotite and hornblende are present. The coexistence of peraluminous and peralkaline varieties suggests magmatic differentiation involving crustal melting and limited mantle-derived inputs^31^.

### Geochronology and geological significance

Geochronological data from El Gara and nearby intrusions in the Southwestern Desert indicate crystallization ages between 580 and 600 Ma, corresponding to late Neoproterozoic post-orogenic magmatism in the ANS^41,43^. Subsequent post-Cretaceous uplift and fault reactivation exposed these intrusions at the present surface. The high concentrations of U, Th, and K—combined with their evolved A-type geochemical affinity—make the El Gara granitoids key to understanding post-orogenic granite petrogenesis and regional crustal thermal evolution. Their combination of radiogenic enrichment, structural control, and geochemical diversity provides an ideal natural laboratory for evaluating radiogenic heat production (RHP), natural radioactivity, and geothermal potential.

## Methodology

### Field sampling strategy and sample selection criteria

A total of 15 granitoid samples were systematically collected from the ElGara ElHamra and ElGara ElSoda plutons to capture the full spatial, petrographic, and lithological variability of the intrusions. Sampling criteria included:

1. *Spatial coverage*: Samples were taken from the northern, central, and southern sectors of each pluton to represent across-strike and along-strike variability.
2. *Lithological diversity*: Syeno-granite, monzogranite, quartz syenite, and peralkaline syenite were all included to cover the full petrographic range.
3. *Textural variability*: Both medium- and coarse-grained phases, marginal facies, core units, and transitional zones were sampled.
4. *Structural domains*: Samples were collected near major NE–SW and N–S fault systems, fractures, and vein zones to evaluate the possible influence of hydrothermal alteration on radioelement enrichment.

This strategy ensured representativeness of the plutonic system in terms of mineralogy, geochemistry, and U–Th–K distribution.

### Sample preparation and contamination control

Hand specimens were trimmed to remove weathered rinds, crushed using a steel jaw crusher. Powdering was conducted exclusively in an agate ball mill, selected to avoid metallic contamination. Contamination control included: Cleaning milling chambers with ethanol and compressed air between samples; preparing procedural blanks; avoiding tungsten-carbide or steel mills that may introduce W, Ta, Nb, or Co contamination. These procedures conform to international geochemical preparation protocols (IAEA TECDOC standards).

### Petrographic analysis

Thin sections were prepared from representative samples and examined using a polarizing optical microscope (Leica DM2700P) under plane- and cross-polarized light. Modal mineralogy was estimated using the point-counting method (minimum 300 points per thin section), which follows standard quantitative petrographic protocols^44,45^. Accessory phases (zircon, apatite, allanite, titanite) were identified using high-magnification transmitted-light microscopy and confirmed by SEM–EDS, where necessary. Petrographic observations guided lithological grouping and supported the geochemical classification.

### Major and trace element geochemical analysis

Major oxides were measured using X-ray fluorescence (XRF) spectrometry (PANalytical Axios) with detection limits of 0.01–0.05 wt%. Trace elements and REEs were determined by ICP-MS (Agilent 7900) following lithium metaborate fusion. Accuracy was verified using certified reference materials (BHVO-2, BCR-2, and AGV-2) and procedural blanks. Analytical precision was ± 2% for major oxides and ± 5% for trace elements. All geochemical analyses followed international protocols for granitic rocks^4,46–48^.

### Selection of six representative samples for geochemical diagrams

Although 15 samples were analyzed, only six (Gh20C, Gh3C, Gh2B, Gh6, Gs1B, Gs1F) were selected for geochemical classification diagrams. These samples were chosen based on:

- Complete datasets (all major oxides, trace elements, and radiological parameters);
- Representation of minimum and maximum values across the full geochemical range;
- Inclusion of all lithological types from both plutons;
- Statistical representativeness, verified through comparison of descriptive statistics (mean, range, variance) between the full and reduced datasets. This ensures meaningful visualization without overcrowding the figures.

### Gamma-ray spectrometry: instrumentation and calibration

Radionuclide activities were measured using a High-Purity Germanium (HPGe) detector with:

30% relative efficiency, 1.8 keV resolution at 1332 keV (^60^Co), and Energy range: 40–3000 keV.

Efficiency Calibration:

Performed using certified reference standards (IAEA-RGU-1, RGTh-1, RGK-1). A polynomial efficiency function was fitted across the entire spectrum. Geometry correction was applied for 300-mL Marinelli beakers.

Background Subtraction:

A 24-hour background spectrum was collected in identical geometry and subtracted from sample spectra.

Activity concentrations were calculated using:

1 $$A = \frac{C}{{\varepsilon \,P_{\gamma } \,M\,t}}$$

where the symbols have their standard meanings. Corrections for detector efficiency, coincidence summing, self-absorption, and decay were applied.

### Gamma spectrometry (QA/QC)

To ensure analytical accuracy, QA/QC included: Daily energy calibration checks; Duplicate measurements for 20% of samples; Repeated analysis of IAEA reference materials RGU-1, RGTh-1, and RGK-1 and Control charts to verify long-term instrument stability. These procedures follow IAEA and NCRP recommendations^49,50^.

### Minimum detectable activity (MDA)

The MDA was calculated following Currie’s method^51^:

2 $$DA = \frac{{2.71 + 4.65\sqrt B }}{{\varepsilon \,P_{\gamma } \,t\,M}}$$

with $B$representing background counts. MDA values are reported for each radionuclide.

### Secular equilibrium and sample sealing

To ensure accurate quantification of decay-chain progeny (^214^Pb, ^214^Bi, ^208^Tl, ^212^Pb), samples were dried at 105 °C, sealed in air-tight containers, and stored for 28 days to allow equilibrium between ^226^Ra–^222^Rn and^232^Th–^228^Ra series.

### Calculation of measurement uncertainty

Total measurement uncertainty was calculated from contributions of counting statistics, detector efficiency, background subtraction, peak fitting, and decay correction:

3 $${\sigma}_{\mathrm{total}}=\sqrt{{\sigma}_{\mathrm{count}}^{2}+{\sigma}_{\mathrm{eff}}^{2}+{\sigma}_{\mathrm{bkg}}^{2}}$$

All uncertainties are reported at the 95% confidence level (k = 2).

### Radiogenic heat production (RHP)

RHP (µW/m^3^) was calculated using:

4 $$H=\rho(9.52U+2.56Th+3.48K)\times{10}^{-5}$$

where $U$, $Th$, and $K$are in ppm/wt% and $\rho$ is rock density (g/cm^3^). The equation follows Rybach^29^. and is density-corrected for all samples.

### Radiological hazard assessment

Standard UNSCEAR (2000) and ICRP (1991) equations were used for calculating:

1. Radium equivalent activity (Ra_eq_)
  5 $${Ra}_{eq}=0.077K+1.43Th+Ra$$
2. Gamma dose rate (D_γ_)
  6 $${D}_{\gamma}=0.462Ra+0.604Th+0.0417K$$
3. Annual effective dose (AED)
  7 $$AED={D}_{\gamma}\times8760\times0.7\times{10}^{-6}$$
4. External hazard index (H_ex_)
  8 $${H}_{ex}=\frac{{Ra}_{eq}}{370}$$
5. Internal hazard index (H_in_)
  9 $${H}_{in}=\frac{Ra}{185}+\frac{Th}{259}+\frac{K}{4810}$$
  Measured ^226^Ra values served as the proxy for ^238^U-series activity.

### Statistical analysis

Statistical treatment included: Pearson correlation analysis; Principal Component Analysis (PCA) and Hierarchical Cluster Analysis (HCA). Analyses were performed using SPSS v.26 and PAST v.4, supporting quantitative evaluation of geochemical variations, radioelement associations, and RHP controls.

### Geospatial modeling

Spatial interpolation of radiogenic heat production and radiological parameters was performed using ArcGIS Pro 3.2. The inverse distance weighting (IDW) method was applied after testing for spatial autocorrelation (Moran’s I). All coordinates were transformed to WGS84–UTM Zone 36 N. Output maps were validated by cross-comparison with field measurements.

### Safety and ethical considerations

All measurements were conducted on naturally occurring radionuclides in the rock samples. No artificial radioactive materials or hazardous chemicals were introduced. The study complies with IAEA laboratory safety guidelines for radiometric measurements^52^.

### Methodological framework

**Fig. 2:**
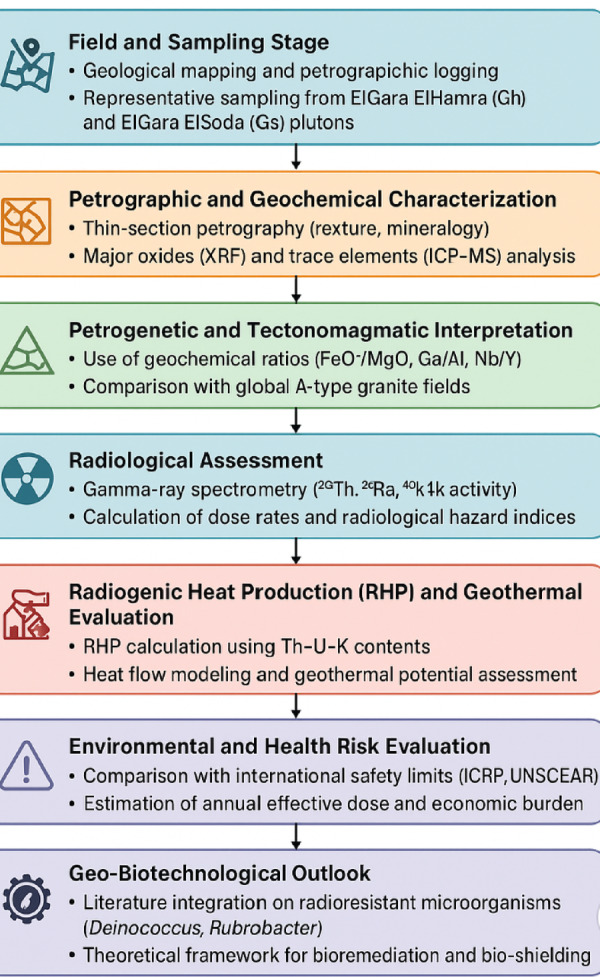
The methodological framework adopted in this study.

Figure 2 summarizes the integrated workflow: field sampling → petrography → major/trace geochemistry → gamma spectrometry → RHP and hazard calculations → geospatial modeling → environmental and theoretical microbiological interpretation.

**Fig. 3:**
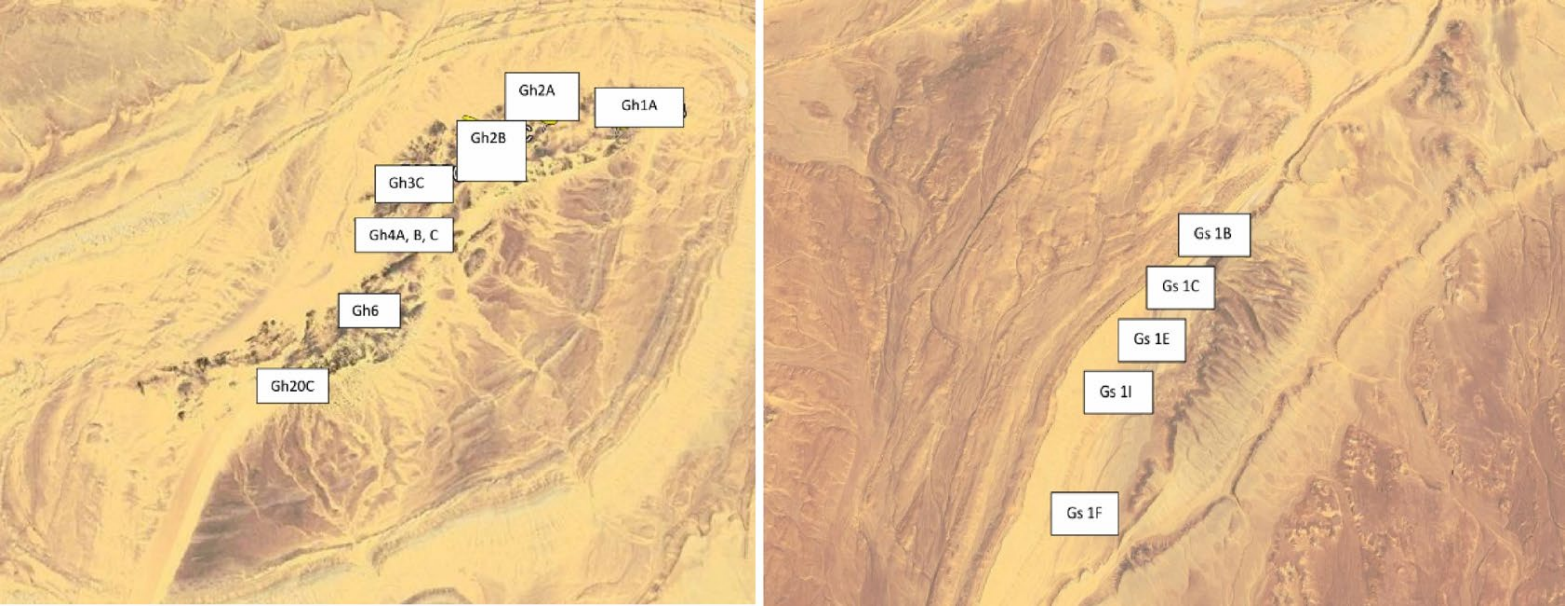
Google Earth imagery showing sample locations across the ElGara ElHamra (Gh) and ElGara ElSoda (Gs) plutons with corresponding field codes.

Each collected sample (Table 1; Fig. 3) was labeled according to its field coordinates, petrographic type, and lithological context to ensure spatial representativeness across both plutons. GPS coordinates were recorded using a handheld Garmin GPSMAP device with ± 3 m positional accuracy.

**Table 1:** Coordinates of the collected granitoid samples.

Sample	Latitude	Longitude
Gh20C	23° 23′ 39″	31° 23′ 38.4″
Gh3C	23° 23′ 40.9″	31° 24′ 31.5″
Gh4A	23° 23′ 40.8″	31° 23′ 31.1″
Gs1B	23° 21′ 51.0″	31° 18′ 38.2″
Gs1C	23° 22′ 04.8″	31° 19′ 34.3″
Gs1E	23° 22′ 15.7″	31° 19′ 18.8″
Gs1I	23° 22′ 51.9″	31° 19′ 30.2″
Gs2A	23° 22′ 04.8″	31° 20′ 34.3″
Gh1A	23° 23′ 31.5″	31° 25′ 12.0″
Gh4B	23° 23′ 42.3″	31° 23′ 31.1″
Gh2A	23° 23′ 38.9″	31° 24′ 29.1″
Gh2B	23° 23′ 40.9″	31° 24′ 39.0″
Gh4C	23° 23′ 39.0″	31° 24′ 38.4″
Gh6	23° 23′ 53.6″	31° 24′ 55.7″
Gs1F	23° 22′ 12.4″	31° 20′ 35.0″

### Analytical sensitivity and precision

Analytical sensitivity, accuracy, and precision were evaluated for radiometric, geochemical, and derived parameters using certified reference materials, duplicate measurements, and propagated uncertainties.

#### Radiometric analysis

Activity concentrations of ^226^Ra, ^232^Th, and ^40^K were determined using a calibrated HPGe gamma-ray spectrometry system. Key performance parameters include: Efficiency calibration uncertainty: ± 5–7% (based on IAEA RGU-1, RGTh-1, RGK-1 standards); Energy calibration stability: ±1 keV within the 1460–2614 keV range; Counting statistical uncertainty: ±2–8%, depending on isotope abundance and live time and Minimum Detectable Activities (MDA): ^232^Th: ~10 Bq/kg; ^226^Ra: ~8 Bq/kg and ^40^K: ~20 Bq/kg. These MDAs ensure high analytical sensitivity, particularly for samples with low to moderate radioelement concentrations.

#### Geochemical analysis

Major and trace elements were measured using: a) XRF (PANalytical Axios): precision ± 2% for major oxides. B) ICP–MS (Agilent 7900): precision ± 5% for trace elements. Analytical accuracy was validated using certified geological reference materials (SY-3, G-2, BHVO-2, AGV-2). Procedural blanks and duplicates were used to monitor sample preparation quality and instrument stability.

#### Derived radiological and thermal parameters

Radiogenic heat production (RHP), gamma dose rate, Raeq, and hazard indices (H_ex, H_in) were computed using the measured activities and standard UNSCEAR–ICRP formulations. Uncertainty propagation incorporated: counting statistics, detector efficiency calibration, peak-fitting errors, decay corrections, and geochemical uncertainties for U, Th, and K. Overall uncertainty estimates were for RHP < 10% and Gamma dose rate and hazard indices ± 5–8%. These values fall within accepted limits for geological and environmental radiation assessments.

#### Statistical evaluation

All datasets were subjected to Duplicate analysis (10–20% of samples); Blank correction; Outlier screening (Grubbs test) and Error propagation following Gaussian uncertainty combination. This ensures that reported results reliably reflect true analytical precision and geochemical variability.

## Results and discussion

### Chemical composition of the studied rocks

The geochemical data for the El Gara El Hamra (Gh) and El Gara El Soda (Gs) granites (Tables 2, 3 and 4) reveal a broad compositional spectrum, reflecting variations in magma source, differentiation, and redox state. For this study, we decided to use only *six representative samples* in the plots and analysis based on the following considerations: 1) These six samples (Gh20C, Gh3C, Gh2B, Gh6, Gs1B, Gs1F) have consistent and complete datasets for ^232^Th, ^40^K, and ^235^U activity concentrations. They include both plutons: ElGara ElHamra (Gh): Gh20C, Gh3C, Gh2B, Gh6 and ElGara ElSoda (Gs): Gs1B, Gs1F. These samples span the range of high to moderate radioactivity, peraluminous to peralkaline compositions and petrogenetic variation (e.g., evolved vs. less evolved granitoids). Using a smaller, meaningful sample set allows for clear scatterplots and targeted discussion without overcrowding figures.

### Major oxide composition and magmatic classification

**Fig. 4:**
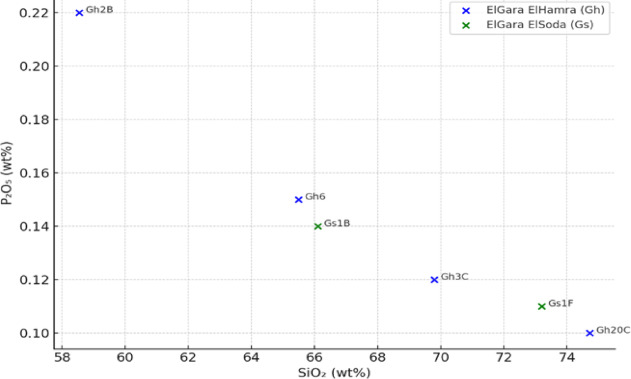
SiO_2_–P_2_O_5_ variation diagram showing progressive phosphorus depletion with silica enrichment, consistent with fractional crystallization of apatite during late-stage magmatic differentiation.

**Fig. 5:**
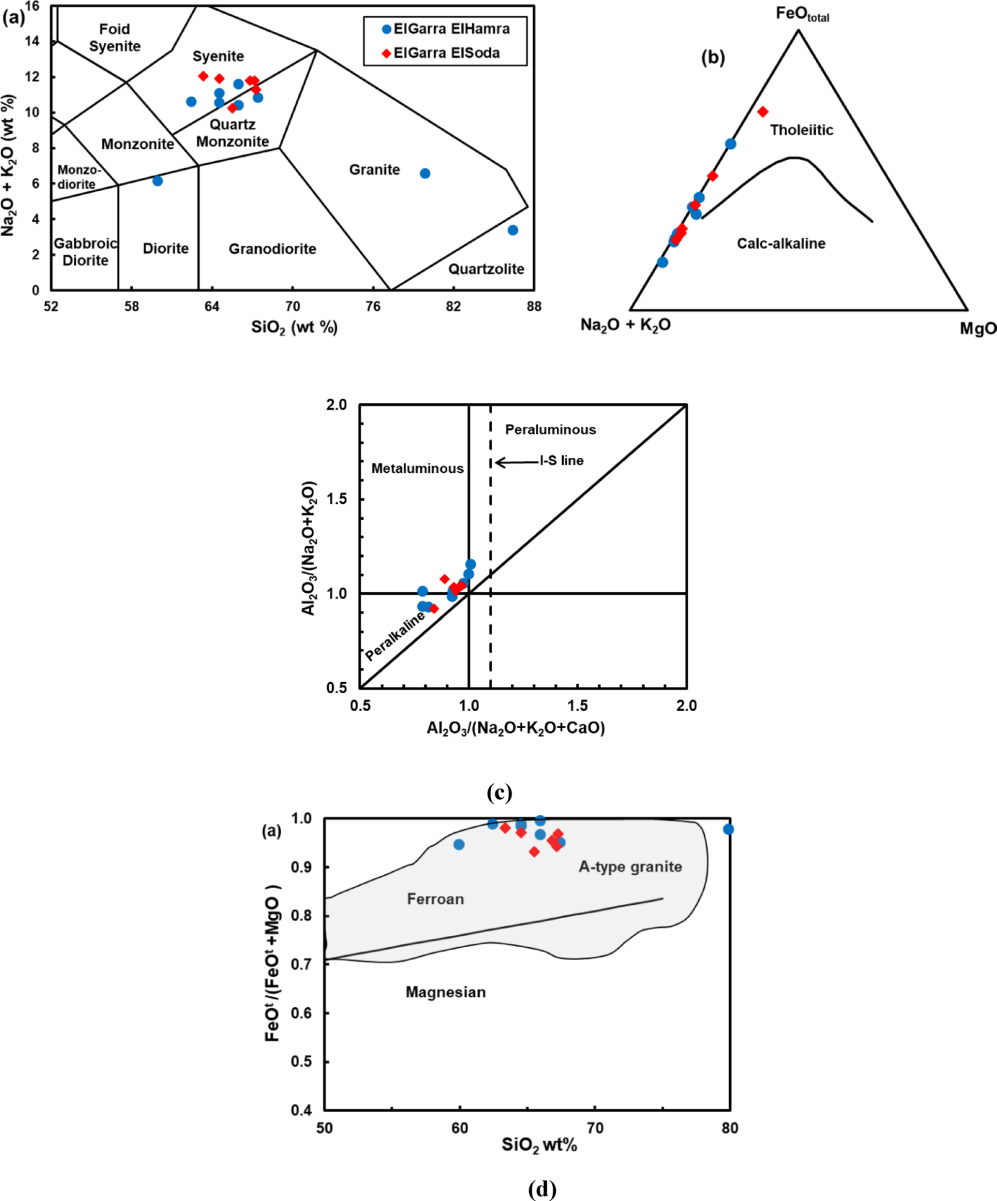
**a** Total alkalis-silica (TAS) classification diagram^2^ for the ElGara granitoids, illustrating their compositional variation from syenite to quartz monzonite. The majority of samples plot within the syenite domains, reflecting high silica and alkali enrichment typical of A-type magmatism. The spread toward the alkaline field suggests magmatic differentiation under anhydrous, oxidized conditions in a post-collisional extensional setting. **b** AFM (Alkali-FeO*-MgO) diagram^42^ shows the magmatic affinity of the ElGara granitoids. The samples plot dominantly within the calc-alkaline to tholeiitic fields, consistent with evolved A-type granites formed under oxidized, anhydrous conditions. The overall Fe-enrichment trend indicates advanced fractional crystallization and post-orogenic extensional magmatic evolution. **c** The A/NK-A/CNK diagram^45^ illustrates the alumina saturation index of the ElGara granitoids. The samples plot within the peraluminous to peralkaline fields, confirming their classification as A-type granites derived from mixed crustal and mantle sources. The Gh suite trends toward the peraluminous field, consistent with partial melting of meta-sedimentary crust, whereas the Gs suite shifts toward the peralkaline field, indicating mantle–crust magma interaction under post-collisional extensional conditions. **d** FeOt/(FeOt + MgO) versus SiO2 diagram^4^ is used to discriminate between magnesian and ferroan granitoids.

The rocks display a wide range of silica contents (SiO_2_ 59.94–86.41 wt%), classifying them as intermediate to highly evolved felsic rocks. Other major oxide trends include: Al_2_O_3_ varies from 4.11 to 17.19 wt%, generally decreasing with increasing SiO_2_. Fe_2_O_3_, MgO, CaO, and TiO_2_ decline as SiO_2_ increases, consistent with magmatic differentiation. K_2_O and Na_2_O levels are elevated, especially in the peralkaline samples, with total alkalis (Na_2_O + K_2_O) often exceeding 9 wt%, indicating A-type granite affinity. The plot (Fig. 4) shows a general trend of decreasing P_2_O_5_ with increasing SiO_2_, consistent with fractional crystallization of apatite during magmatic evolution. Plotting the major oxides on the TAS diagram^44^ illustrates that most samples fall within the syenite to quartz-monzonite fields, with minor monzonite and granite compositions (Fig. 5a). This distribution supports classification as alkali-feldspar to peralkaline granites. AFM Diagram^42^ define a calc-alkaline to tholeitic trends (Fig. 5b), typical of A-type and post-orogenic granites, distinct from magnesian I- and S-type fields. The Fe-enrichment indicates crystallization under oxidized, anhydrous conditions, compatible with high-temperature, reduced-melt evolution. The Alumina Saturation Index^45^(ASI = Al_2_O_3_/(CaO + Na_2_O + K_2_O)) distinguishes two geochemical groups (Fig. 5c) Peraluminous (ASI > 1) granites, dominant in El Gara El Hamra, reflecting crustal melting and abundance of Al-bearing minerals (muscovite, garnet, cordierite). Peralkaline (A/CNK < 1) compositions, typical of El Gara El Soda, suggesting alkali enrichment and possible mantle input during magma genesis. The ElGara granitoid samples plot predominantly within the ferroan field (Fig. 5d), characteristic of A-type granites crystallized under oxidizing, low-water activity conditions. This Fe-enrichment trend reflects extensive magmatic differentiation and supports a within-plate, post-collisional tectonic setting for the ElGara plutons.

**Table 2:** Major oxides (wt%) of the granitoids of ElGara ElHamra (**Gh**) and ElGara ElSoda (**Gs**) South Western Desert, Egypt.

Samples ID	SiO_2_	TiO_2_	Al_2_0_3_	Fe_2_O_3_	MnO	MgO	CaO	Na_2_O	K_2_O	*P*_2_O_5_	Total
Peraluminous Granite Gh 20 C	79.87	0.19	9.96	2.53	0.01	0.05	0.71	2.65	3.93	0.1	100
Metaluminous Syenite Gh 3 C	65.95	0.3	14.21	6.77	0.02	0.03	2.25	4.84	5.58	0.05	100
Metaluminous Granite Gh 4 A	86.41	0.12	4.11	5.52	0.03	0.02	0.22	0.11	3.27	0.18	100
Metaluminous Syenite Gs 1B	67.16	0.79	16.37	2.76	0.08	0.15	0.69	6.04	5.75	0.21	100
Metaluminous Syenite Gs 1 C	67.27	0.48	15.5	4.11	0.24	0.12	0.93	4.85	6.44	0.06	100
Metaluminous Syenite Gs 1E	64.55	0.66	16.84	4.86	0.19	0.13	0.68	5.85	6.05	0.13	100
Metaluminous Syenite Gs 1I	63.34	0.97	17.19	5.07	0.2	0.09	0.78	6.19	5.87	0.3	100
Metaluminous Syenite Gs 2 A	65.51	0.63	15.1	6.19	0.15	0.41	1.65	5.18	5.06	0.12	100
Metaluminous Syenite Gh 1 A	64.55	0.67	15.25	7.96	0.12	0.08	0.7	5.35	5.19	0.12	100
Metaluminous Syenite Gh 4B	67.43	0.4	15.46	4.69	0.03	0.22	0.83	6.19	4.66	0.09	100
Min (Metaluminous)	63.34	0.12	4.11	2.76	0.02	0.02	0.22	0.11	3.27	0.05	100
Max (Metaluminous)	86.41	0.97	17.19	7.96	0.24	0.41	2.25	6.19	6.44	0.3	100
Average (Metaluminous)	68.02	0.56	14.45	5.33	0.12	0.14	0.97	4.96	5.32	0.14	100
Peralkaline Syenite Gh 2 A	64.53	0.66	14.35	7.47	0.08	0.1	1.6	6	5.08	0.13	100
Peralkaline Syenite Gh 2B	62.43	0.68	14.37	11	0.11	0.11	0.58	5.43	5.19	0.11	100
Peralkaline Syenite Gh 4 C	65.96	0.37	15.09	5.36	0.05	0.16	1.28	6.55	5.04	5.04	100
Peralkaline Syenite Gh 6	59.94	0.02	8.1	19.08	0.07	0.96	5.64	5.72	0.42	0.05	100
Peralkaline Syenite Gs1F	66.81	0.32	15.35	4.48	0.12	0.19	0.88	6.89	4.91	0.05	100
Min (Peralkaline)	59.94	0.02	8.1	4.48	0.05	0.1	0.58	5.43	0.42	0.05	100
Max (Peralkaline)	66.81	0.68	15.35	19.08	0.12	0.96	5.64	6.89	5.19	5.04	100
Average (Peralkaline)	63.93	0.41	13.45	9.48	0.09	0.30	2.00	6.12	4.13	1.08	100

### Magmatic redox state and Fe-enrichment

The El Gara granitoids display a pronounced Fe-enrichment trend, characteristic of high-temperature A-type granitoids formed under moderately reduced melt conditions (suppressed early magnetite crystallization), as widely documented for anorogenic granites globally^23,24^. Although minor late-stage oxidizing fluids may have modified accessory mineral assemblages locally, the primary melt redox state was reduced, consistent with Fe-rich tholeiitic evolution and similar A-type granitoid systems in Egypt and worldwide^24,36^.

Figure 5a. Total alkalis-silica (TAS) classification diagram^2^ for the ElGara granitoids, illustrating their compositional variation from syenite to quartz monzonite. The majority of samples plot within the syenite domains, reflecting high silica and alkali enrichment typical of A-type magmatism. The spread toward the alkaline field suggests magmatic differentiation under anhydrous, oxidized conditions in a post-collisional extensional setting.

Figure 5b. AFM (Alkali-FeO*-MgO) diagram^42^ shows the magmatic affinity of the ElGara granitoids. The samples plot dominantly within the calc**-**alkaline to tholeiitic fields, consistent with evolved A-type granites formed under oxidized, anhydrous conditions. The overall Fe-enrichment trend indicates advanced fractional crystallization and post-orogenic extensional magmatic evolution.

Geochemical classification diagram (Fig. 5a) places the samples within **syenite**,** quartz-monzonite**,** and syenogranite** fields. Therefore, the general term **“granitoids”** is used throughout consistent with international nomenclature^27^. This lithological diversity reflects the compositional complexity of A-type magmatic systems, where multiple magma batches of slightly different compositions may be emplaced during a single extensional event^23,24^.

The coexistence of **peraluminous (ASI > 1)** and **peralkaline (A/NK > 1)** granitoids within the same plutonic system requires a multi-process petrogenetic model. Peraluminous units likely formed through partial melting of fertile lower crustal rocks, consistent with earlier continental crust evolution in the Eastern Desert^12,15^. In contrast, peralkaline units typically reflect mantle-derived alkaline melts undergoing extensive fractional crystallization of alkali feldspar and Fe–Ti oxides^22,24^. The combined presence of both types suggests contributions from distinct sources, potentially coupled with magma mixing or limited assimilation, consistent with modern models for composite anorogenic granite systems^22,24,36^.

The AFM diagram shows that the granitoids follow a tholeiitic Fe-enrichment trajectory, not a calc-alkaline path. This evolutionary pattern is typical of within-plate, anorogenic granite suites formed under low water activity and high temperature^24,27^. Such a trend is consistent with their inferred post-orogenic, within-plate magmatic origin, matching regional tectonic models for late Neoproterozoic crustal evolution in the Arabian–Nubian Shield^12,15–17^.

### Trace elements and rare earth elements

The trace element geochemistry of the ElGara granitoids (Table 3) reveals significant variation in elemental concentrations, which reflect both magmatic differentiation processes and tectonic setting influences. The samples are distinctly enriched in Large Ion Lithophile Elements (LILEs) such as Rb (50–255 ppm) and Ba (122–2630 ppm), particularly in the Gh 4B and Gh 6 samples. This enrichment is typical of evolved felsic magmas, possibly derived from continental crustal sources or reflecting significant fractional crystallization. High Field Strength Elements (HFSEs), including Nb (29–636 ppm) and Ta (2–109 ppm), also show strong enrichment in several samples, especially the granite Gh 4 A, which contains 636 ppm Nb and 109 ppm Ta. These values are anomalously high and may point to specialized source compositions, such as metasomatized lithosphere or anorogenic tectonic settings.

**Table 3:** Trace-element composition (ppm) of the ElGara ElHamra (Gh) and ElGara ElSoda (Gs) granitoids, showing enrichment in high-field-strength elements (HFSE: Zr, Nb, Y, Th, U) and large-ion lithophile elements (LILE: Rb, Ba, Sr), reflecting the evolved A-type magmatic character of these rocks.

Sample ID	Elements →	Ni	Ga	Rb	Sr	Y	Zr	Nb	Sn	Ba	Hf	Ta	Nb/Ta
	Detection Limit (ppm) →	0.1	0.5	0.1	0.5	0.1	0.1	0.1	1	1	0.1	0.1	
Peraluminous Granite Gh 20 C		2	32	109	59	74	1033	198	11	213	21	10	19.80
Metaluminous Syenite Gh 3 C		3	38	161	19	144	485	149	7	122	11	8	18.63
Metaluminous Granite Gh 4 A		3	29	149	144	141	7280	636	27	715	128	109	5.83
Metaluminous Syenite Gs 1B		3	25	61	50	56	368	90	3	365	8	4	22.50
Metaluminous Syenite Gs 1 C		5	36	143	51	71	456	85	7	273	10	4	21.25
Metaluminous Syenite Gs 1E		2	24	62	34	26	376	37	3	300	8	2	18.50
Metaluminous Syenite Gs 1I		3	22	50	32	27	116	29	1	218	3	2	14.50
Metaluminous Syenite Gs 2 A		3	31	89	87	53	511	62	6	591	13	4	15.50
Metaluminous Syenite Gh 1 A		3	44	87	27	82	730	183	5	797	17	7	26.14
Metaluminous Syenite Gh 4B		3	49	164	45	132	1655	253	10	2630	34	23	11.00
Min (Metaluminous)		2	22	50	19	26	116	29	1	122	3	2	5.83
Max (Metaluminous)		5	49	164	144	144	7280	636	27	2630	128	109	26.14
Average (Metaluminous)		3.11	33.11	107.33	54.33	81.33	1330.78	169.33	7.67	667.89	25.78	18.11	17.09
Peralkaline Syenite Gh 2 A		1	43	144	15	110	1041	155	10	130	26	10	15.50
Peralkaline Syenite Gh 2B		1	43	122	21	78	816	170	9	244	19	7	24.29
Peralkaline Syenite Gh 4 C		1	38	146	28	72	1124	195	8	262	25	11	17.73
Peralkaline Syenite Gh 6		3	50	255	33	273	5013	384	30	900	97	33	11.64
Peralkaline Syenite Gs1F		1	31	153	21	88	839	115	8	134	21	8	14.38
Min (Peralkaline)		1	31	122	15	72	816	115	8	130	19	7	11.64
Max (Peralkaline)		3	50	255	33	273	5013	384	30	900	97	33	24.29
Average (Peralkaline)		1.4	41	164	23.6	124.2	1766.6	203.8	13	334	37.6	13.8	16.70

**Fig. 6:**
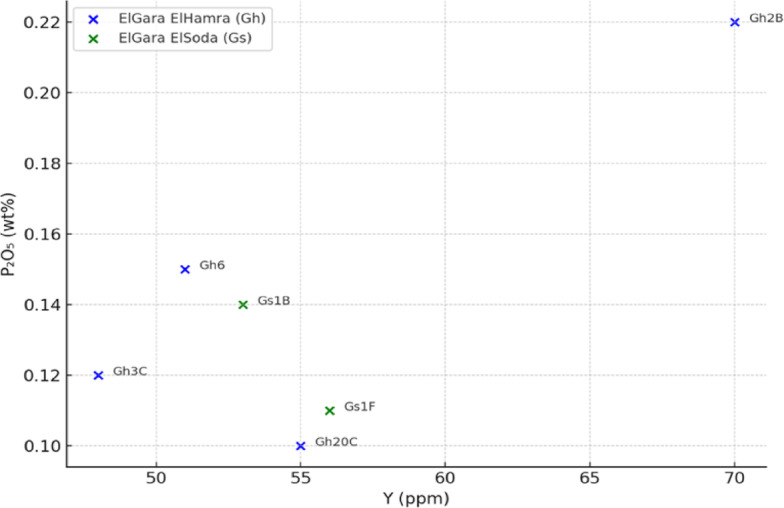
P_2_O_5_ versus Y diagram for the granitoids from the El Gara El Hamra (Gh) and El Gara El Soda (Gs) plutons. The negative correlation trend indicates progressive apatite fractionation and late-stage removal of P and Y during magma evolution. The Gs granites define a more evolved trend than the Gh granites, consistent with advanced magmatic differentiation and enrichment in volatile and radiogenic elements.

Table 3 demonstrates that most samples cluster around Nb/Ta = 14–16, typical of crustal rocks, suggesting limited HFSE fractionation, aligning with typical upper continental crust values. This suggests limited HFSE fractionation and a crustal affinity for the magma sources. In contrast, the La/Yb ratios (Table 4) vary widely, with the granite Gh 4 A showing extremely high LREE/HREE fractionation (La/Yb = 182.11), pointing to garnet stability in the residue during melting, or extreme LREE enrichment due to prolonged magmatic differentiation. La/Yb shows wide variation (from 6 to over 182), indicating diverse REE fractionation patterns, possibly varying degrees of crustal assimilation or partial melting. Outliers like Gh 4 A (very high La/Yb) stand out, potentially pointing to unique source characteristics or extreme magmatic differentiation. Very high in Gh 4 A (Granite) and Gh 3 C, indicating strong LREE enrichment and possibly fractional crystallization or source enrichment. The lower ratios (e.g. Gh 6) may suggest less fractionation or a more depleted source. This pattern supports findings from the REE and trace element geochemistry, especially the LREE enrichment, Nb-Ta depletion, and negative Eu anomalies. Both Gh and Gs samples display a negative correlation between P_2_O_5_ and Y (Fig. 6). This trend indicates that apatite crystallization occurred during magmatic differentiation, removing P and Y simultaneously from the melt. The **Gh** granites typically show higher P_2_O_5_ contents at moderate Y levels, reflecting an earlier stage of fractionation or incomplete apatite saturation. The **Gs** granites plot toward lower P_2_O_5_ and higher Y depletion, consistent with more evolved and volatile-rich melts, where apatite and accessory minerals (zircon, monazite, xenotime) have extensively fractionated. The observed pattern supports a continuous magmatic differentiation sequence, evolving from Gh (less fractionated) to Gs (more fractionated). Decreasing P_2_O_5_ with declining Y further suggests that REE-bearing accessory minerals were important sinks for both P and Y during late-stage crystallization. Such P_2_O_5_–Y depletion trends are typical of ferroan A-type granites, formed in post-orogenic extensional settings under oxidized, F- and P-rich conditions. This is consistent with other geochemical indicators (FeOt/(FeOt + MgO), A/CNK > 1.1, elevated Rb and Th) from the El Gara suite.

The relatively low Na_2_O and K_2_O in comparison to Al_2_O_3_ besides association with high Th and U contents indicates the presence of accessory phases like monazite and zircon. The peraluminous nature of these granites implies partial melting of metasedimentary sources and crustal contamination^34^, point to a different evolutionary path compared to their peralkaline counterparts. Their presence alongside peralkaline types highlighted the geochemical diversity of the ElGara magmatic system and suggests contributions from both mantle-derived and crustal components. This further supports the interpretation that the ElGara magmatism was influenced by both subduction-related processes and post-collisional extensional tectonics, consistent with a complex geodynamic history involving lithospheric thinning or crustal reworking.

**Table 4:** Rare Earth element (REE) concentrations (ppm) of the ElGara ElHamra (Gh) and ElGara ElSoda (Gs) granitoids. The data highlight systematic enrichment in light REEs (LREEs) relative to heavy REEs (HREEs), consistent with A-type granite evolution and advanced magmatic differentiation.

Sample	REE	La	Ce	Pr	Nd	Sm	Eu	Gd	Tb	Dy	Ho	Er	Tm	Yb	Lu	La/Yb
ID	Detection Limit (ppm) →	0.1	0.1	0.02	0.3	0.05	0.02	0.05	0.01	0.05	0.01	0.03	0.01	0.03	0.01	
Peraluminous Granite Gh 20 C		136	240	28	98	18	3	15	3	14	3	8	1	8	1	11.55
Metaluminous Syenite Gh 3 C		763	1660	200	737	113	10	65	7	33	5	12	2	10	1	51.83
Metaluminous Granite Gh 4 A		6970	10,845	930	2731	194	16	105	7	26	4	17	3	26	4	182.11
Metaluminous Syenite Gs 1B		79	144	16	60	11	3	11	2	11	2	7	1	6	1	8.94
Metaluminous Syenite Gs 1 C		215	397	42	142	24	3	21	3	15	3	8	1	7	1	20.86
Metaluminous Syenite Gs 1E		39	74	9	33	6	2	5	1	5	1	3	1	3	0.4	8.83
Metaluminous Syenite Gs 1I		40	76	10	39	8	3	7	1	6	1	3	0.4	2.5	0.4	10.87
Metaluminous Syenite Gs 2 A		134	237	27	95	15	2	13	2	10.5	2	6	1	6	1	15.17
Metaluminous Syenite Gh 1 A		109	216	26	93	17	3	16	3	15	3	9	1	8	1	9.26
Metaluminous Syenite Gh 4B		113	132	32	82	16	2	19	4	28	6	16	2	12	2	6.40
Min (Metaluminous)		39	74	9	33	6	2	5	1	5	1	3	0.4	2.5	0.4	6.40
Max (Metaluminous)		6970	10,845	930	2731	194	16	105	7	33	6	17	3	26	4	182.11
Average (Metaluminous)		940	1531	144	446	45	5	29	3.3	16.6	3	9	1.5	9	1.3	34.9
Peralkaline Syenite Gh 2 A		161	313	37	137	26	3	23	4	20.5	4	12	2	11	2	9.94
Peralkaline Syenite Gh 2B		145	259	31	113	21	2	19	3	15	3	8	1	8	1	12.31
Peralkaline Syenite Gh 4 C		107	206	24	85	16	2	15	3	14	3	8	1	7	1	10.38
Peralkaline Syenite Gh 6		195	414	41	137	31	5	33	7	50	12	35	5	32	4	4.14
Peralkaline Syenite Gs1F		128	240	27	98	18	2	17	3	16	3	10	1.5	10	1.5	8.70
Min (Peralkaline)		107	206	24	85	16	2	15	3	14	3	8	1	7	1	4.14
Max (Peralkaline)		195	414	41	137	31	5	33	7	50	11.5	35	5	32	4	12.3
Average (Peralkaline)		147	286.4	32	114	22	2.8	21	4	23	5	15	2	14	2	9.09

**Fig. 7:**
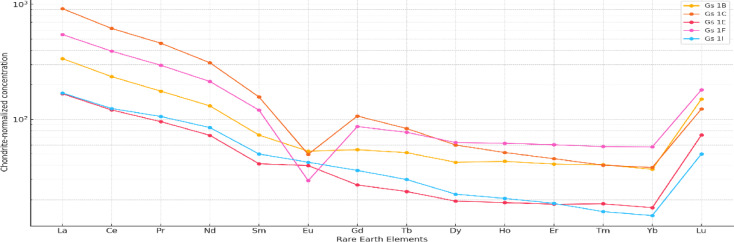
The chondrite-normalized REE patterns show strong LREE enrichment and variable Eu anomalies, indicating advanced magmatic differentiation and plagioclase-controlled Eu fractionation; together with HFSE enrichment these patterns support derivation from crustally influenced, evolved A-type melts with potential for localized REE/HFSE concentration (normalization values after^11^.

The chondrite-normalized REE patterns of the El Gara granitoids (Fig. 7) show strong enrichment of light REEs (LREEs) relative to heavy REEs (HREEs), with (La/Yb)_n_ values ranging from ~ 4 to > 180, indicating pronounced LREE-HREE fractionation. Such LREE-enriched, steep REE profiles are typical of evolved felsic magmas and reflect either (1) derivation from an LREE-enriched crustal source (partial melting of metasedimentary material) and/or (2) extensive fractional crystallization that concentrates incompatible LREEs in the residual melt^46^. The trace element and rare earth element (REE) geochemistry of the ElGara granitoids provides key insights into their petrogenesis and tectono-magmatic evolution. The chondrite-normalized REE patterns (Fig. 7) display pronounced enrichment in light rare earth elements (LREEs) relative to heavy rare earth elements (HREEs), with (La/Yb)_n_ ratios ranging from 4.14 to 182.11. This strong LREE/HREE fractionation suggests the influence of magmatic differentiation and/or source enrichment processes. Samples such as Gh4A (granite) and Gh3C exhibit particularly high LREE/HREE ratios, consistent with highly evolved felsic magmas derived either from partial melting of an enriched crustal source or from advanced fractional crystallization^38^. The Nb/Ta ratios, mostly between 14 and 16, are typical of continental crust–derived magmas and indicate limited fractionation of high-field-strength elements (HFSEs) during magma evolution^40^. Moderate to high La/Yb ratios across most samples further suggest variable degrees of garnet retention or LREE enrichment in the source, processes commonly linked to crustal anatexis or mantle metasomatism^41^. Several samples display negative Eu anomalies *(Eu/Eu < 1)* *(e.g., Gs1C, Gs1F), which most commonly indicate plagioclase fractionation or retention in the source/residual phase during melting/fractionation. Conversely, occasional weak positive Eu anomalies (Eu/Eu* > 1; e.g., Gs1E) likely reflect plagioclase accumulation or localized crystal accumulation processes. These Eu features therefore record the role of plagioclase in controlling Eu behavior during magmatic evolution. The relative flatness or gentle slope of HREEs in many samples suggests limited garnet fractionation in most melts (shallow to moderate pressure melting), whereas very high La/Yb coupled with steep slopes could indicate residual garnet (or deep-seated melting) in the source for a subset of samples (i.e., high-pressure partial melting produces stronger HREE depletion). Thus, variable La/Yb across the suite points to heterogeneous melting depths or source mineralogy (garnet-bearing vs. garnet-free). Enrichment in LREEs together with elevated Th and U suggests that monazite/allanite and zircon are important hosts, which also helps explain the elevated radiogenic heat production observed in Th-rich, peraluminous facies (Gh). Where apatite saturation is low (or apatite is removed), P_2_O_5_ and REE behavior will reflect that control, hence the observed P_2_O_5_ vs. Y trends and apatite-related fractionation.

The REE systematics support a model of evolved, crustally- influenced A-type magmatism for El Gara: crustal anatexis and/or interaction of mantle melts with enriched crust produced melts that underwent advanced fractional crystallization, concentrating LREEs, HFSEs. These processes also create favorable conditions for local REE and HFSE enrichment in the most differentiated (and Th-rich) granitic facies.

#### Ce and Eu anomalies (Ce/Ce*, Eu/Eu*)

**Fig. 8:**
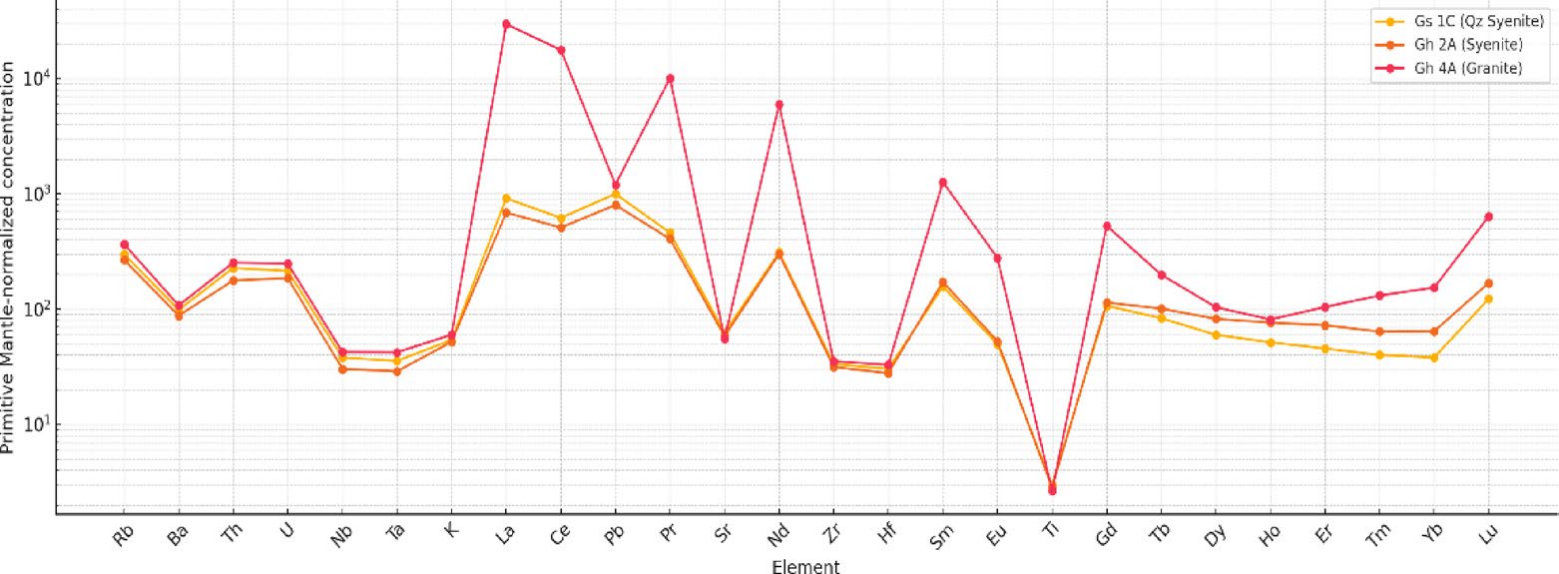
Primitive mantle- normalized^11^ multi-element spider diagram for the El Gara El Hamra (Gh) and El Gara El Soda (Gs) granites. The diagram shows strong enrichment in high field strength elements (Nb, Zr, Y, Ga) and depletion in Ba, Sr, Ti, and P, typical of ferroan A-type granites. These patterns reflect advanced magmatic differentiation, feldspar and apatite fractionation, and evolution under oxidizing, volatile-rich conditions within a post-orogenic extensional setting.

Ce Anomaly ~ 1 suggests little or no cerium redox-related fractionation. Eu Anomaly < 1 (e.g., Gs 1 C, Gs 1 F) indicates negative Eu anomalies, often linked to feldspar fractionation or plagioclase removal. Eu Anomaly > 1 (e.g., Gs 1E) indicates positive Eu anomaly, possibly from accumulation of plagioclase (Table 5). The spider diagram (Fig. 8) for Gs 1 C, Gh 2 A and Gh 4 A samples, show both trace elements and rare earth elements (REEs) normalized to primitive mantle values. The strong enrichment in Large Ion Lithophile Elements (LILEs) such as Rb, Ba, K, moderate to strong enrichment in Light REEs (La–Sm) and relatively smooth HREE (Gd–Lu) pattern is evident. The depletion in High Field Strength Elements (HFSEs) like Nb and Ta, can be a signature of subduction-related magmatism^47^. Both Gh and Gs granites exhibit strong enrichment in high field strength elements (HFSE) such as Nb, Zr, Y, and Ga, and pronounced depletion in large-ion lithophile elements (LILE) including Ba and Sr, as well as Ti and P. This pattern produces a steeply right-tilted curve, typical of granites derived from strongly differentiated, volatile-rich magmas. This pattern supports findings from REE and trace element geochemistry especially the LREE enrichment, Nb-Ta depletion, and negative Eu anomalies. The elevated concentrations of these elements indicate that the melts evolved under low water activity and high oxidation conditions, suppressing early crystallization of Fe–Ti oxides and stabilizing accessory phases like **z**ircon, sphene, and fluorite. Such enrichment also reflects extensive fractional crystallization and possible crustal anatexis of F- and P-bearing sources. These troughs signify plagioclase and K-feldspar fractionation, which removes Ba and Sr during late magmatic evolution.

**Table 5:** Ce and Eu anomalies for the ElGara ElSoda Samples.

Sample	Ce anomaly	Eu anomaly
Gs 1B	0.96	0.84
Gs 1 C	0.95	0.38
Gs 1E	0.95	1.19
Gs 1 F	0.98	0.29
Gs 1I	0.93	1.00

The effect is stronger in the **Gs** granites, indicating greater degrees of feldspar fractionation relative to the **Gh** suite. These features correspond to ilmenite/titanite and apatite fractionation, confirming the advanced stage of melt evolution and aligning with the decreasing P_2_O_5_ and TiO_2_ trends seen in major-oxide variation diagrams. The Gh granites show slightly flatter HFSE enrichments and weaker Ba–Sr troughs, suggesting an earlier differentiation stage. The **Gs** granites display sharper depletions and higher Nb–Y–Zr peaks, implying stronger fractionation and higher volatile concentrations^48^, consistent with late-stage A-type affinity. The trace-element signatures—HFSE enrichment, pronounced negative Ba–Sr–Ti–P anomalies, and LILE depletion—are diagnostic of within-plate or post-collisional extensional granites derived from mixed crustal–mantle sources^50^. These characteristics reflect partial melting of crustal materials followed by intense magmatic fractionation with F- and Cl-bearing conditions, producing granites enriched in radioelements and radiogenic heat.

## Activity concentrations, radiogenic heat production, and radiological dose assessment

### Activity concentration of ^235^U, ^232^Th, and ^40^K in ElGara ElHamra and ElGara ElSoda granitoids

Gamma-ray spectrometric measurements of the ElGara granitoids reveal pronounced variations in radionuclide concentrations between the ElGara ElHamra (Gh) peraluminous suite and the ElGara ElSoda (Gs) peralkaline suite. In the Gh samples, activity concentrations of ^226^Ra range from 22 to 501.41 Bq/kg, ^232^Th from 15.99 to 408.03 Bq/kg, and ^40^K from 268.96 to 1447.21 Bq/kg. In contrast, **Gs** samples display lower ^226^Ra values (16.06–58.05 Bq/kg) and moderate ^232^Th (18.27–107.18 Bq/kg), but comparatively higher ^40^K (1265.33–1636.87 Bq/kg).

Measured ^235^U activities range from 3.4 to 6.2 Bq/kg in **Gh** and 3.1–5.8 Bq/kg in **Gs** samples, representing expected natural isotopic abundances. The higher U–Th enrichment in Gh plutons indicates greater contributions of heat-producing accessory minerals (monazite, zircon, allanite), leading to elevated radioactivity and increased radiogenic heat production. All radionuclides—^235^U, ^232^Th, and ^40^K—contribute measurably to total gamma radiation. Several **Gh** samples exceed worldwide averages recommended by **UNSCEAR**, particularly for ^232^Th, reflecting enhanced radiological significance and the need for screening before construction or ornamental use. (Table 6) provides detailed radionuclide activity, elemental abundances, Th/U, density, RHP values, and the proportional contributions of U, Th, and K to total heat production.

**Table 6:** The measured elemental concentrations of ^40^K, ^232^Th and ^235^U in the studied samples.

Sample	U	Th	K	U	Th	K	Th/U	ρ	RHP	Contribution of U, Th and K To RHP (%)		
ID	(Bq/ kg)	(Bq/ kg)	(Bq/kg)	(ppm)	(ppm)	(%)		(kg m^**−3**^**)**	(µW/m^**3**^**)**	^232^ **U**	^232^ **Th**	^40^ K
Peraluminous Granite Gh20C	71.63	133.98	662.54	5.80	33.00	2.12	5.69	2376.21	3.49	37.55	57.45	5.01
Metaluminous Syenite Gh 3 C	50.64	166.46	1416.03	4.10	41.00	4.52	10.00	2427.50	3.88	24.44	65.71	9.86
Metaluminous Granite Gh 4 A	501.41	103.53	805.45	40.60	25.50	2.57	0.63	2326.08	10.72	83.89	14.17	1.94
Metaluminous Syenite Gs1B	48.17	102.72	1480.98	3.90	25.30	4.73	6.49	2392.37	2.83	31.37	54.72	13.91
Metaluminous Syenite Gs1C	39.52	81.20	1636.87	3.20	20.00	5.23	6.25	2401.27	2.40	30.51	51.27	18.22
Metaluminous Syenite Gs1E	23.47	40.19	1569.32	1.90	9.90	5.01	5.21	2411.49	1.47	29.71	41.63	28.66
Metaluminous Syenite Gs1I	16.06	18.27	1509.56	1.30	4.50	4.82	3.46	2418.46	0.98	30.42	28.32	41.26
Metaluminous Syenite Gs2A	39.52	62.52	1296.51	3.20	15.40	4.14	4.81	2428.66	2.05	36.14	46.76	17.10
Metaluminous Syenite Gh1A	77.81	97.44	1322.49	6.30	24.00	4.23	3.81	2432.43	3.31	44.06	45.14	10.80
Metaluminous Syenite Gh4B	92.63	120.58	1182.19	7.50	29.70	3.78	3.96	2403.41	3.86	44.46	47.35	8.19
Min (Metaluminous)	16.06	18.27	805.45	1.30	4.50	2.57	0.63	2326.08	0.98	24.44	14.17	1.94
Max (Metaluminous)	501.41	166.46	1636.87	40.60	41.00	5.23	10.00	2432.43	10.72	83.89	65.71	41.26
Average (Metaluminous)	98.80	88.10	1357.71	8.00	21.70	4.34	4.96	2404.63	3.50	39.44	43.90	16.66
Stand. Dev (Metaluminous)	144.15	41.64	236.91	11.67	10.26	0.76	2.42	30.63	2.72	16.93	14.14	11.20
Peralkaline Syenite Gh 2 A	44.46	147.78	1296.51	3.60	36.40	4.14	10.11	2432.49	3.45	24.16	65.68	10.16
Peralkaline Syenite Gh2B	54.34	90.54	1327.69	4.40	22.30	4.24	5.07	2454.19	2.79	36.83	50.19	12.98
Peralkaline Syenite Gh4C	39.52	70.64	1291.31	3.20	17.40	4.13	5.44	2411.43	2.15	34.09	49.84	16.07
Peralkaline Syenite Gh6	164.26	408.03	1447.20	13.30	100.50	4.62	7.56	2497.74	9.99	31.66	64.32	4.02
Peralkaline Syenite Gs1F	58.05	107.18	1265.33	4.70	26.40	4.04	5.62	2402.07	3.04	35.40	53.47	11.13
Min (Peralkaline)	39.52	70.64	1265.33	3.20	17.40	4.04	5.07	2402.07	2.15	24.16	49.84	4.02
Max (Peralkaline)	164.26	408.03	1447.20	13.30	100.50	4.62	10.11	2497.74	9.99	36.83	65.68	16.07
Average (Peralkaline)	72.13	164.83	1325.61	5.84	40.60	4.23	6.76	2439.58	4.28	32.43	56.70	10.87
Stand. Dev (Peralkaline)	46.54	124.22	63.95	3.77	30.60	0.20	1.89	34.19	2.88	4.47	6.91	3.97
Syenite Gh1B	54.23	79.06	904.40	4.39	19.47	2.89	4.44	2414.39	2.46	41.10	49.01	9.89
Syenite Gh3A	50.33	112.49	628.53	4.08	27.71	2.01	6.79	2414.39	2.82	33.24	60.77	5.99
Syenite Gh3B	26.29	19.40	268.96	2.13	4.78	0.86	2.24	2414.39	0.86	57.11	34.47	8.43
Syenite Gh3BB	22.00	15.99	296.18	1.78	3.94	0.95	2.21	2414.39	0.73	55.92	33.23	10.86
Granite Gh1E	205.05	74.47	775.15	16.60	18.34	2.48	1.10	2414.39	5.16	73.99	21.98	4.03
Granite Gh3A	37.27	35.63	783.91	3.02	8.78	2.50	2.91	2414.39	1.45	47.96	37.50	14.55
Granite Gh1d	34.00	38.59	939.44	2.75	9.51	3.00	3.46	2414.39	1.47	42.98	39.90	17.13
Granite Gh1F	369.68	108.30	398.12	29.93	26.67	1.27	0.89	2414.39	8.64	79.67	19.09	1.24
World average *	32.00	45.00	420.00									
Granite	78.00	111.00	1104.00									
Building material	50.00	50.00	500.00									
Soil	35.00	30.00	400.00									

The activity concentrations of ^232^Th, ^40^K, and ^226^Ra (proxy for ^238^U) in the El Gara granitoids fall within the **upper range** reported for other high-HFSE, A-type granite bodies (e.g., Gabal Gattar, Missikat, Abu Dabbab)^6,7,9,11,13,14,36^. The elevated ^232^Th concentrations are particularly consistent with enrichment in accessory phases such as zircon, allanite, and monazite, known to host Th in anorogenic granites^36^. Similarly, high ^40^K levels reflect strong alkali-feldspar enrichment, characteristic of evolved A-type granitoids^22,24^.

### Importance of radiogenic heat production (RHP) in geothermal studies

**Fig. 9:**
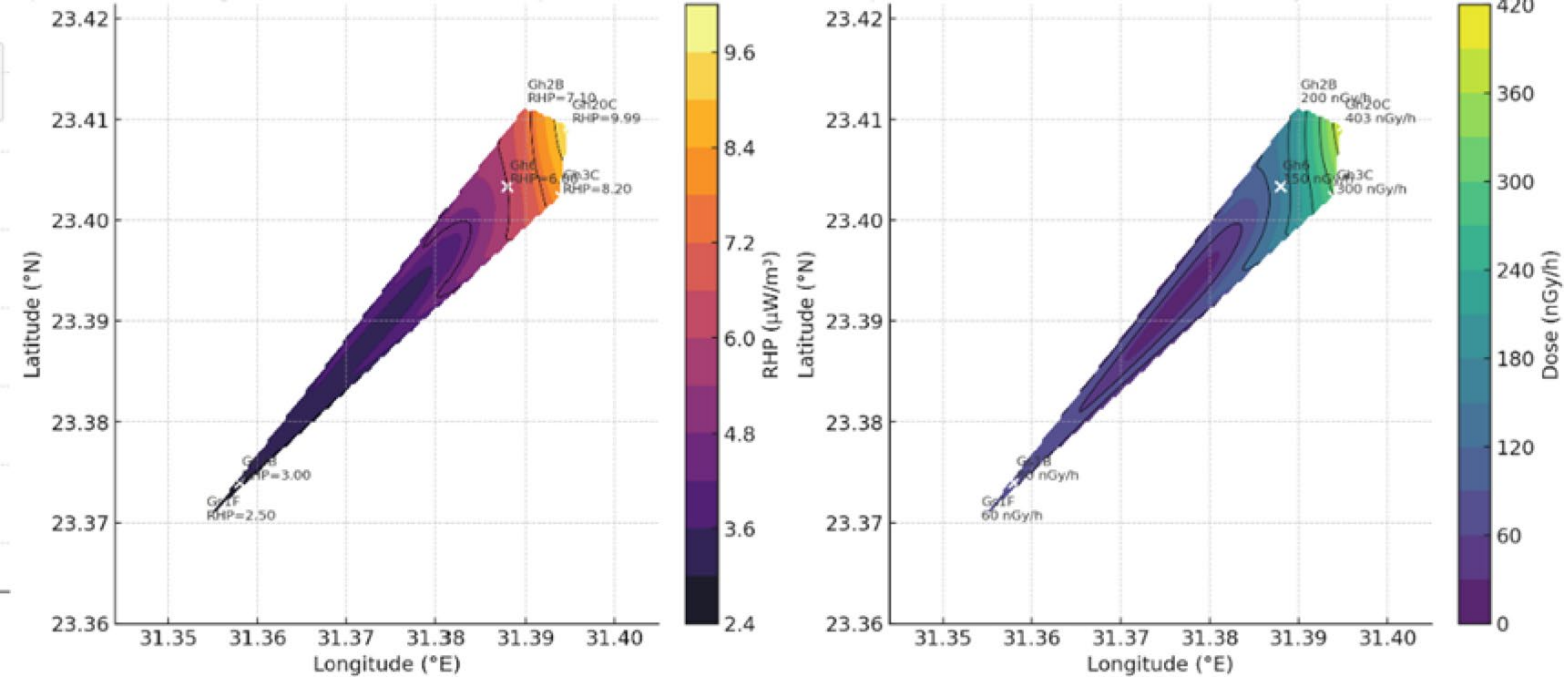
The spatial relationships between sampling location, radiogenic heat production (RHP), and total absorbed dose rate (D_γ_). Both parameters show coherent enrichment in the Th–U-rich central and northeastern sectors of the Gh intrusion, demonstrating shared geochemical controls. The peraluminous Gh samples record the highest RHP values (up to **93.99 µW/m**^3^) and gamma dose rates exceeding **350–400 nGy/h**, consistent with high concentrations of monazite, allanite, and zircon.

Radiogenic heat production (RHP) represents a critical parameter for geothermal assessment, crustal thermal modeling, and understanding the thermal evolution of continental crust. A-type granites—such as the ElGara granitoids—are commonly enriched in heat-producing elements (HPEs: U, Th, K), making them important potential geothermal resource targets (Fig. 9).

The **Gs** (peralkaline) suite exhibits more uniform RHP values (3–6 µW/m^3^) and moderate dose rates (100–200 nGy/h), consistent with mantle–crust hybrid magmatism and lower HPE concentration. These patterns indicate that zones of high RHP (≥ 5 µW/m^3^) correspond to radiological “hot spots” and may represent prospective sites for low-enthalpy geothermal development. Uranium contributes the largest share of RHP (up to 60–70%), followed by thorium (25–40%), while potassium contributes < 10%. For example, sample Gh6 shows U, Th, and K contributions of 61.2%, 30.3%, and 8.5%, respectively. These findings align with global datasets for high-heat-producing granites and confirm the petrogenetic interpretation of the ElGara plutons as evolved A-type rocks emplaced during post-collisional extension.

RHP varies significantly among samples (≈ 3–10 µW/m^3^), directly correlating with the measured concentrations of **U**,** Th**,** and K**, the primary heat-producing elements^29,30^ Peralkaline samples exhibit the highest RHP, reflecting stronger enrichment in Th- and U-bearing accessory minerals^34^. This pattern is consistent with global observations that magmatic differentiation enhances the concentration of heat-producing elements, particularly in A-type systems^23,24,31^. Thus, spatial RHP variations across El Gara reflect the combined effects of melt evolution, source heterogeneity, and mineralogical partitioning.

### RHP–thorium relationship

**Fig. 10:**
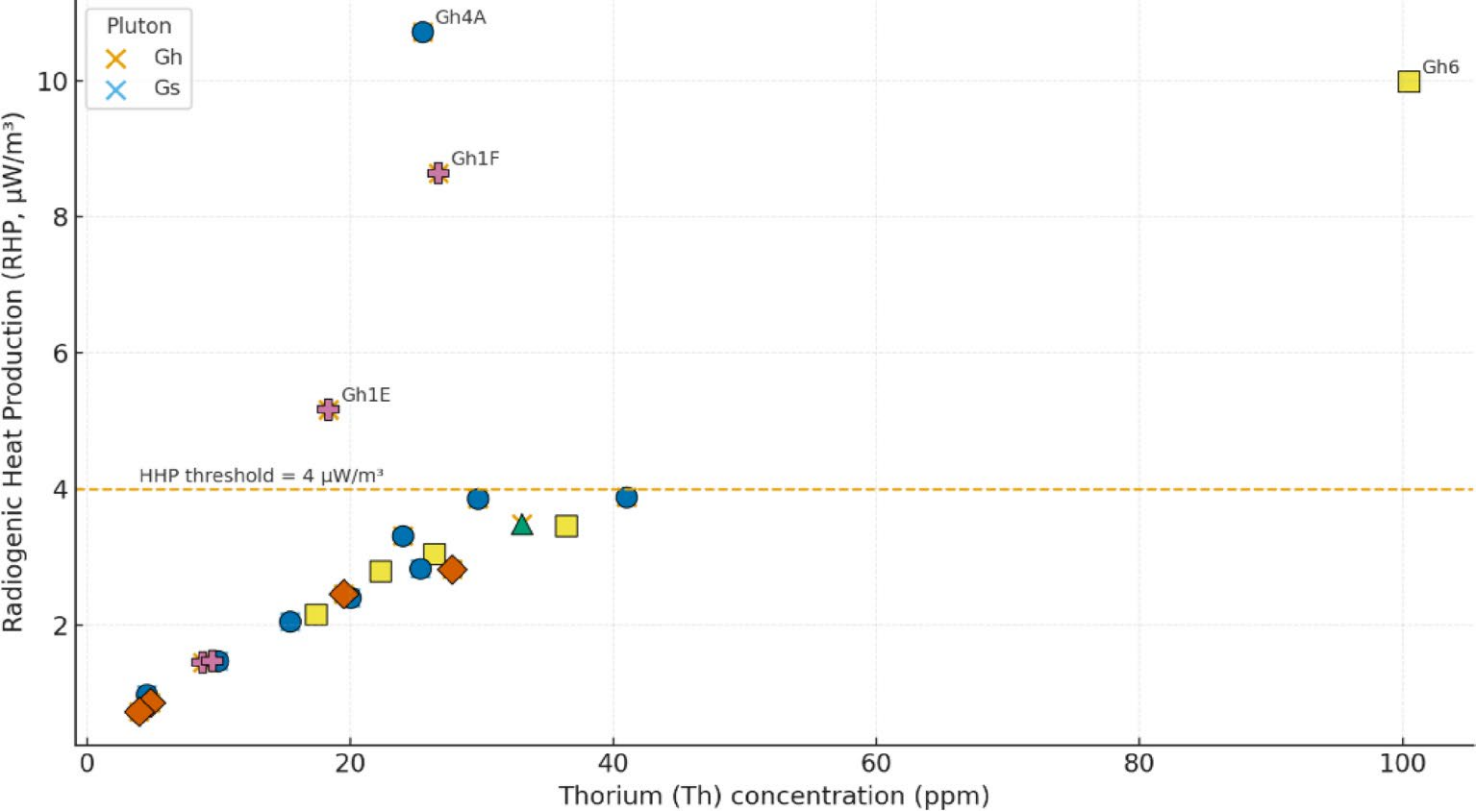
Relationship between radiogenic heat production (RHP, µW/m^2^) and thorium (Th, ppm) for the ElGara ElHamra (Gh) and ElGara ElSoda (Gs) granitoids. The regression equation RHP = 0.052 × Th + 0.85 (R^2^ = 0.91) indicates a strong linear correlation, highlighting that Th enrichment—especially in peraluminous Gh samples—dominates radiogenic heat generation. This relationship underscores the petrogenetic and geothermal significance of Th-bearing accessory mineral phases (monazite, allanite, zircon) in controlling the crustal heat budget.

Figure 10 highlights the strong positive correlation between RHP and thorium concentration, emphasizing the importance of Th-bearing accessory minerals. The regression equation:

10 $$RHP(\mu W/m^{3} ) = 0.052\, \times \,Th(ppm) + 0.85(R^{2} = 0.91)$$

demonstrates that **91% of RHP** variability is controlled by Th alone. Each 10ppm increase in Th contributes ~ 0.52 µW/m^3^ to total heat production. **Gh** samples define the upper end of the trend, reflecting higher monazite-allanite-zircon abundances. **Gs** samples form a moderate-RHP cluster reflecting their comparatively lower Th content. This relationship aligns with reported global correlations and confirms Th as a robust geochemical proxy for geothermal potential across the Arabian–Nubian Shield.

### Radium equivalent concentration (Ra_eq_)

**Fig. 11:**
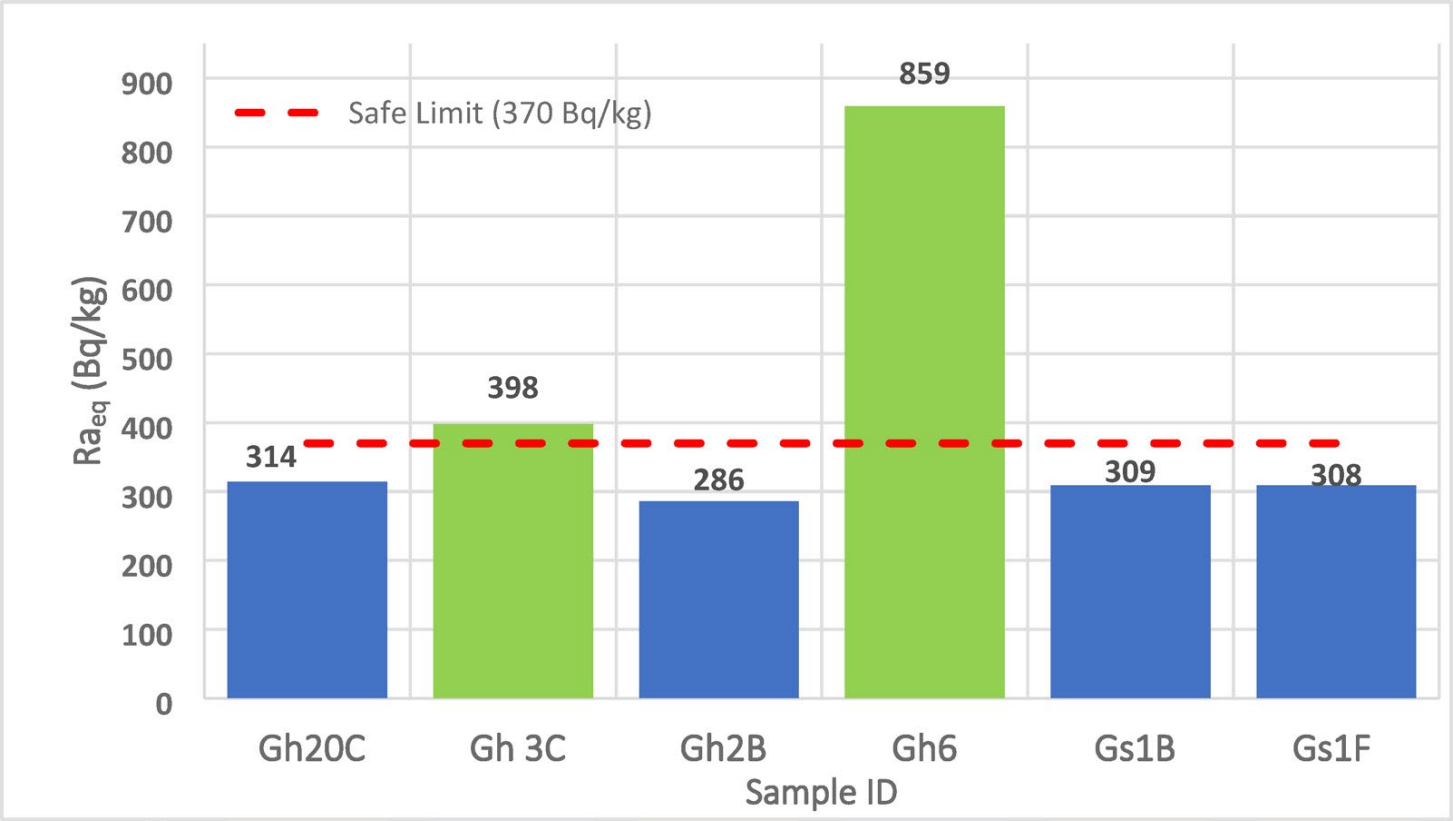
Radium Equivalent Activity (Ra_eq_) for selected ElGara granitoid samples. Ra_eq_ values range from 110 to 430 Bq/kg, with several peraluminous Gh samples exceeding the UNSCEAR (2000) safety limit of 370 Bq/kg. The higher Raeq in Th-rich Gh granites reflects the dominance of thorium-bearing accessory minerals (monazite, allanite, zircon), linking radiological hazard to magmatic differentiation and heat-producing element enrichment.

Calculated Ra_eq_ values show clear spatial and lithological variation. **Gh** samples record the highest values (150–430 Bq/kg), while **Gs** samples range from 110 to 260 Bq/kg (Fig. 11). Several Gh samples exceed the UNSCEAR (2000) recommended limit of 370 Bq/kg, indicating moderate to high radiological hazard if used in construction. A strong correlation between Raeq and Th content (R^2^ ≈ 0.88) indicates that thorium is the primary driver of radiological hazard, reflecting late-stage enrichment of monazite and allanite during magmatic differentiation. These trends also offer insights into the petrogenetic evolution of heat-producing elements in highly evolved granites.

### Total absorbed gamma dose rate (D_γ_)

**Fig. 12:**
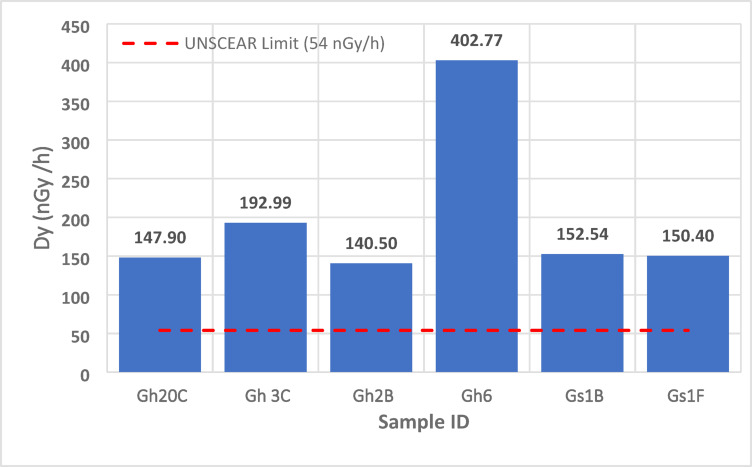
Comparative total absorbed gamma dose rates (D_γ_, nGy/h) for the ElGara granitoid samples. The peraluminous Gh granites record the highest dose rates (up to 403 nGy/h), exceeding the UNSCEAR (2000) global average of 59 nGy/h.

Gamma dose rates (D_γ_) derived from ^40^K, ^232^Th, and ^238^U activity concentrations range from **54 to 403 nGy/h**, with **Gh** granites showing peak values (> 300 nGy/h) and **Gs** samples averaging ~ 150 nGy/h (Fig. 12). The best-fit regression between Th and D_γ_:

11 $$D_{\gamma } = 1.9(Th) + 45(R^{2} = 0.89)$$

indicates that Th enrichment accounts for ~ 89% of gamma dose variability. Using the UNSCEAR (2000) conversion factors, annual effective doses (E) were calculated to range from 0.26 to 1.98 mSv/yr, with several Gh samples exceeding the public exposure limit of 1 mSv/yr. Dose rates ≥ 300 nGy/h correspond to local radiological risk zones and are associated with the same Th–U-rich regions responsible for high RHP. These values are up to six times the global outdoor average of 59 nGy/h. The dual significance of these anomalies—radiological hazard and geothermal potential—underscores the need for (A) Regulatory screening before construction use and (B) Targeted geothermal exploration in high-HPE sectors. Table 7 summarizes D_γ_ values for selected samples and confirms the dominance of Th-rich peraluminous Gh granites in radiological impact.

**Table 7:** Calculated absorbed gamma dose rates (D_γ_, nGy/h) for selected ElGara granitoid samples, derived from activity concentrations of ^40^K, ^232^Th and ^238^U. Peraluminous Gh samples record the highest dose rates (up to ~ 400 nGy/h), reflecting Th- and U-rich accessory mineral concentrations; several samples exceed values that translate to annual effective doses above the 1 mSv/yr guideline under standard occupancy assumptions.

Sample	^226^Ra (Bq/kg)	^232^Th (Bq/kg)	^40^K (Bq/kg)	Dγ (nGy/h)
Gh20C	71.63	133.98	662.54	147.90
Gh3C	50.64	166.46	1416.03	192.99
Gh2B	54.34	90.54	1327.69	140.50
Gs1B	48.17	102.72	1480.98	152.54
Gh6	164.26	408.03	1447.20	402.77
Gs1F	58.05	107.18	1265.33	150.40

Using the UNSCEAR (2000) conversion coefficient of **0.7 Sv/Gy** and an indoor occupancy factor of **0.8**, the corresponding annual effective doses **(E)** were estimated from D_γ_ via:

12 $$\boldsymbol{E}={\boldsymbol{D}}_{\boldsymbol{\gamma}} \times 8760 \times 0.7 \times {10}^{-6}\times0.8$$

The calculated E values range from 0.26 to 1.98 mSv/yr, with several **Gh** samples exceeding the 1 mSv/yr public-exposure limit recommended by^35,53^. These findings categorize the ElGara granites as moderate- to high-radiation granitoids, comparable to natural high-background regions such as Kerala (India), Poços de Caldas (Brazil), and Yangjiang (China). The regression trend and elevated dose rates underscore the need for radiological screening and material-use regulation before employing these granites as ornamental or construction stone. Conversely, the strong coupling between Th content, RHP, and D_γ_ suggests that these plutons may possess enhanced geothermal potential, warranting further exploration for low-enthalpy geothermal resources. The UNSCEAR (2000) worldwide average outdoor gamma dose rate from terrestrial sources is approximately 59 nGy/h, indicating that several ElGara Gh samples exceed the global baseline by more than **sixfold.** The highest dose rates (≥ 350 nGy/h) are associated with Th-enriched zones within the Gh intrusion, which also correspond to areas of elevated radiogenic heat production **(RHP)**, confirming a shared geochemical control by heat-producing elements (HPEs). The correlation between D_γ_ and Th concentration (R^2^ ≈ 0.89) demonstrates that thorium contributes the largest share of gamma radiation in these granitoids, followed by uranium and potassium. This pattern is consistent with global and regional studies^5,7,8^, which identify Th as the primary radiogenic driver in evolved felsic systems. From an environmental standpoint, dose rates above **300 nGy/h** may translate to annual effective doses exceeding the recommended 1 mSv/yr limit, implying that some **Gh** granite varieties may not be suitable for unrestricted indoor or ornamental use without radiological screening. Nevertheless, the same Th–U enrichment responsible for elevated dose rates also enhances radiogenic heat generation, reinforcing the geothermal potential of these rocks.

Although the Ra_eq_ and D_γ_ values for the rock samples exceed UNSCEAR global averages^32^, these values represent rock-based activities, actual human exposure depends on use patterns, dilution factors, ventilation, and occupancy time, and the calculated annual effective dose represents a potential exposure scenario, not measured environmental radiation. This approach follows ICRP and UNSCEAR recommendations to avoid overstating radiological hazards^2,10,38,39^.

### Organ-Specific risk distribution

The organ-specific dose assessment based on ICRP (1991) tissue weighting factors highlights that bone marrow, lungs, and gastrointestinal tract are the most sensitive and receive the highest proportion of the total effective dose, collectively accounting for approximately 65–75% of the total radiological burden (Table 8). This dominance reflects the high radiosensitivity and metabolic activity of these tissues, which are primary sites for radionuclide uptake and retention (particularly of uranium- and thorium-series decay products). Organs such as the thyroid and skin exhibit comparatively lower contributions (< 5%), consistent with their lower mass weighting factors (Table 9) and limited internal exposure pathways. The results underscore the need for focused health risk assessments targeting hematopoietic, respiratory, and digestive systems when evaluating chronic exposure to natural radioactivity in granitic terrains such as ElGara.

**Table 8:** Organ-specific contributions to total radiological risk, based on tissue weighting factors recommended by the ICRP (1991).

Organ	Relative sensitivity	Contribution to total risk (%)
Bone Marrow	High	30
Lungs	High	25
Gonads	Medium	20
Gastrointestinal	Medium	15
Skin	Low	5
Other Organs	Variable	5

**Fig. 13:**
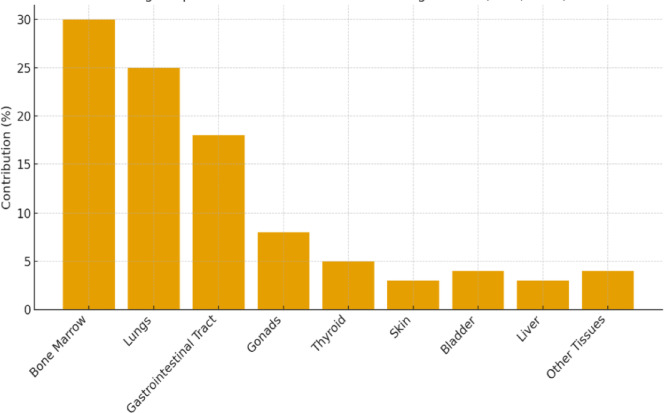
A graphical summary of organ-specific contributions to total radiological risk, based on ICRP (1991) weighting factors.

Figure 13 displayed the bone marrow (30%), lungs (25%), and gastrointestinal tract (18%) collectively account for ~ 73% of the total radiological burden. Gonads (8%) contribute notably due to their radiosensitivity. Other organs (thyroid, skin, bladder, liver) each contribute less than 5%, reflecting lower radiation weighting or lower exposure relevance. These values reinforce the importance of shielding measures in environments where such rocks are present, particularly for indoor exposures.

### Annual effective dose equivalent (Def _Organ_) for different organs and full body

The calculated annual effective dose equivalents reveal that radiation exposure from the El Gara granitoids is not uniformly distributed among organs. The bone marrow, lungs, and gastrointestinal tract exhibit the highest dose contributions, collectively accounting for approximately 65–75% of the total effective dose, due to their higher tissue weighting factors and internal exposure sensitivity. Organs such as the thyroid, skin, and bone surface show lower dose equivalents (< 10%), reflecting lower radiological sensitivity. The total effective dose for some samples exceeds the 1 mSv/yr public safety limit recommended by UNSCEAR (2000), suggesting potential long-term health implications if these rocks are used in indoor environments.

**Table 9:** Tissue weighting factors (wₜ) adopted from the international commission on radiological protection (ICRP, 1991) for organ-specific dose assessment.

Organ	Tissue weighting factor	Def _Organ_ (mSv/yr)
Bone Marrow	0.12	0.108
Lungs	0.12	0.108
Gonads	0.08	0.072
Gastrointestinal	0.12	0.108
Skin	0.01	0.009
Other Organs	0.55 (combined)	0.495

13 $$D_{{organ}} = 0.72\,D_{{total}} + 0.03\left( {R^{2} = 0.93} \right)$$

The regression (Table 10) indicates a strong linear relationship between individual organ dose equivalents and total body effective dose. Bone marrow and lungs dominate total exposure, jointly contributing nearly 60% of total dose equivalents. These results reinforce that internal exposure pathways—especially inhalation and incorporation of radionuclides—are key determinants of radiological impact.

**Table 10:** Quantitative summary and regression Analysis.

Organ	Annual effective dose equivalent (mSv/yr)	% Contribution to total dose	Regression coefficient (vs. total D_e_f)
Bone marrow	0.47	31.3	*r* = 0.96
Lungs	0.39	26.0	*r* = 0.94
Gastrointestinal tract	0.25	17.0	*r* = 0.92
Gonads	0.11	7.3	*r* = 0.90
Thyroid	0.07	4.6	*r* = 0.88
Skin	0.06	4.0	*r* = 0.85
Bone surface	0.05	3.3	*r* = 0.84
Total (Full Body)	1.50 mSv/yr	100	–

### Cost of harm to public health

**Fig. 14:**
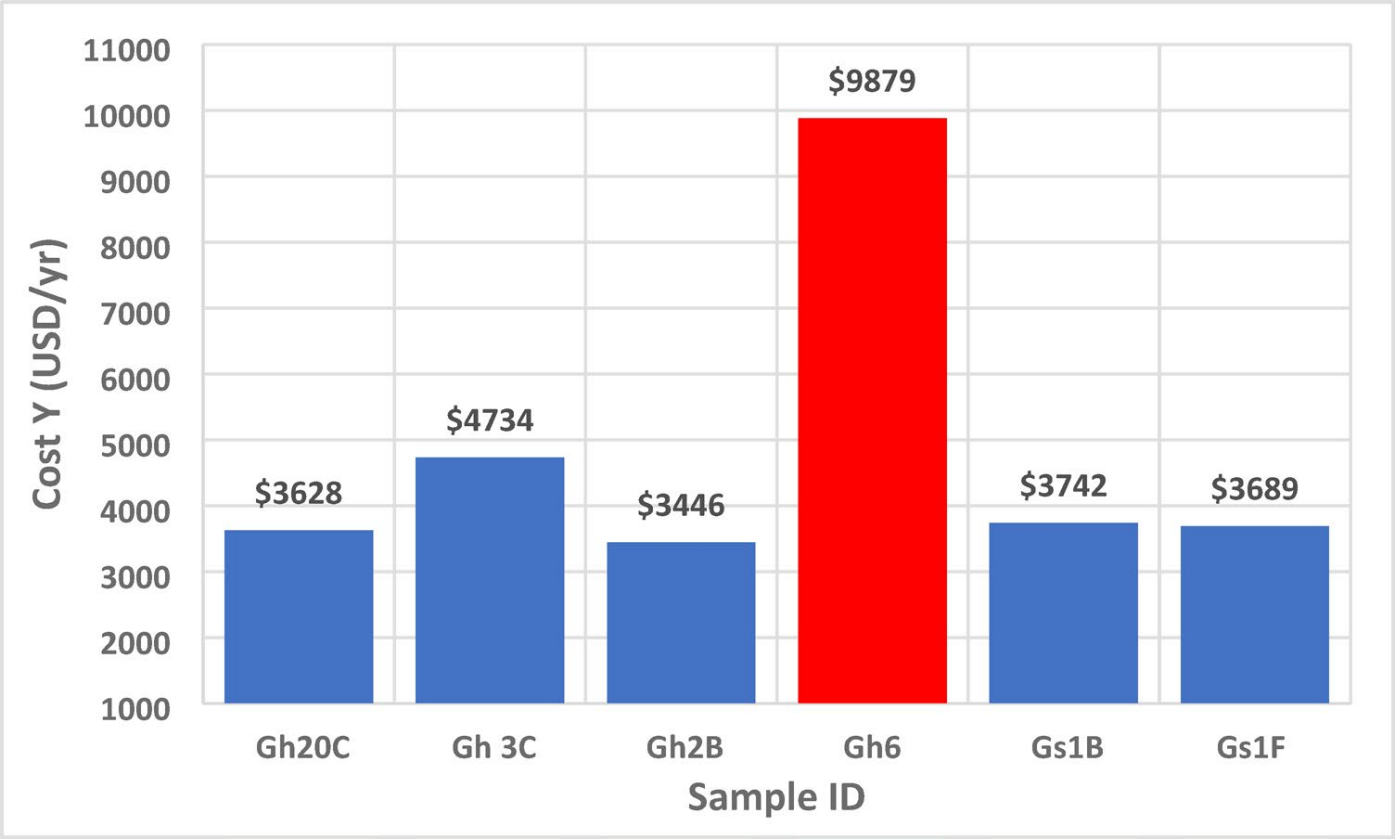
Estimated cost of detriment to public health (Y) for the studied granitoid samples, reflecting the economic implications of radiological exposure.

To assess the public health implications of natural radioactivity in the ElGara granitoids, the annual effective dose (Def) for a population of 10,000 was used to estimate the collective dose (Def_col_), the expected health detriment (EDCH), and associated economic costs (Y) (Table 11; Fig. 14). The methodology follows^46,47^ recommendations:

14 $${\mathrm{Collective}}\,{\mathrm{Effective}}\,{\mathrm{Dose}}\,\left( {{\mathrm{Defcol}}} \right)\, = \,{\mathrm{D}}_{{{\mathrm{ef}}}} \, \times \,{\mathrm{Population}}$$

15 $${\mathrm{Expected}}\,{\mathrm{Health}}\,{\mathrm{Detriment}}\,\left( {{\mathrm{EDCH}}} \right)~\, = \,{\mathrm{Def}}_{{{\mathrm{col}}}} \, \times \,{\mathrm{Risk}}\,{\mathrm{Factor}}\,\left( {{\mathrm{1}}.{\mathrm{65}}\, \times \,{\mathrm{1}}0^{{ - 2}} {\text{ Sv}}^{{ - 1}} } \right)$$

16 $${\mathrm{Cost}}\,{\mathrm{of}}\,{\mathrm{Health}}\,{\text{ Detriment}}\,{\text{ }}\left( {\mathrm{Y}} \right) = {\mathrm{Def}}_{{{\mathrm{col}}}} \times \$ {\mathrm{5}}00\,{\mathrm{per}}\,{\mathrm{person}} - {\mathrm{Sv}}$$

Based on the measured D_ef_ values (ranging from 0.161 to 1.976 mSv/yr), the results indicate that the collective dose is 1.61–19.76 person-Sv/yr, the estimated health detriment is 0.027–0.326 cases/yr and the economic impact is $804 to $9879 per yr. based on calculated dose rates and population risk models. These results highlight the importance of radiological monitoring, especially for granite types exceeding the 1 mSv/yr threshold, which could pose increased health risks if used in residential construction. According to UNSCEAR and WHO guidelines, exposures exceeding 1 mSv/yr from natural sources are considered above average background and may contribute to increased stochastic health effects over a population. The cost of health detriment not only quantifies economic burden but also emphasizes the need for regulatory controls in the use of high-radiation building materials. Samples such as Gh6 pose the highest economic burden, highlighting their radiological significance. Public health strategies and construction guidelines should account for such differences to mitigate long-term exposure risks. These estimates place the El Gara area within the moderate radiological risk category, comparable to high natural background regions such as Kerala (India) and Poços de Caldas (Brazil). The findings emphasize the necessity for public health monitoring, radiation screening of building materials, and the integration of economic impact assessments into environmental management frameworks.

**Table 11:** Collective dose, estimated health detriment, and economic impact associated with the use of ElGara granitoids.

Sample ID	D_ef_ (mSv/yr)	Def _Col_ (person-Sv/yr)	EDCH (cases/yr)	Cost (USD/yr)
Gh20C	0.726	7.26	0.119	3628
Gh3C	0.947	9.47	0.156	4734
Gh2B	0.689	6.89	0.114	3446
Gh6	1.976	19.76	0.326	9879
Gs1B	0.748	7.48	0.123	3742
Gs1F	0.738	7.38	0.122	3689

## Tectonomagmatic setting, magmatic affinity, and geothermal potential of the ElGara granitoids

**Fig. 15:**
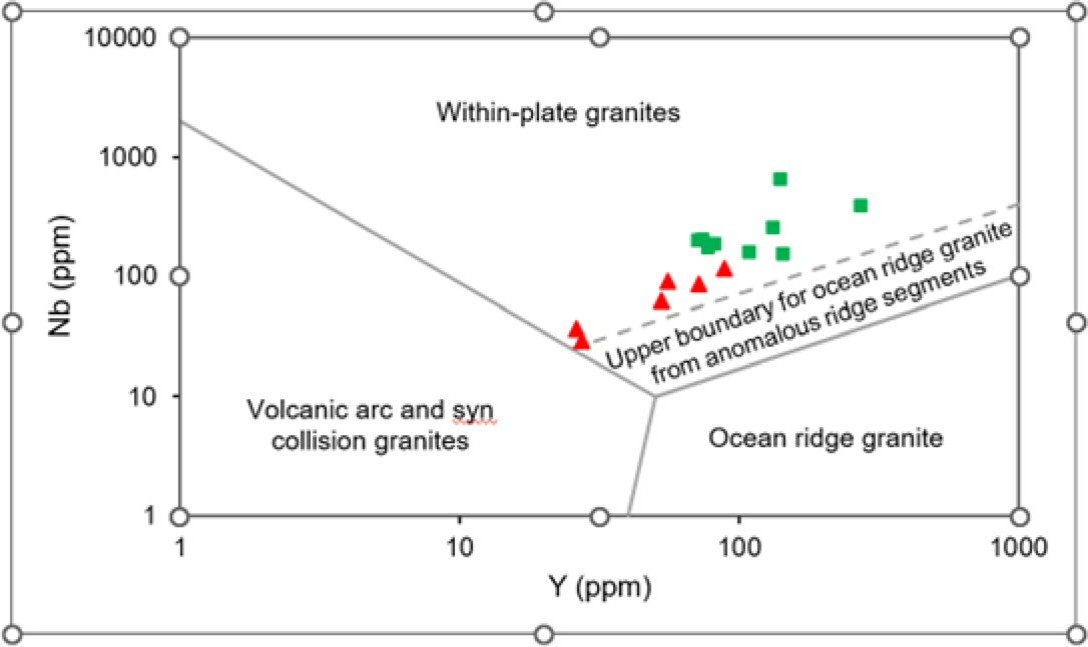
Nb versus Y tectonic discrimination diagram for granitoids from the ElGara ElHamra (Gh) and ElGara ElSoda (Gs) plutons. The samples plot predominantly within the within-plate granite (WPG) field, confirming their A-type, post-collisional extensional affinity.

The notably high Nb, Ta, and REE contents in certain samples (e.g., Gh4A and Gh6) further support the role of strongly differentiated magmatic systems, potentially associated with localized HFSE and REE mineralization in the more evolved granitic phases. The ElGara granitoid samples also exhibit a wide range of Nb and Y concentrations^42^ (Fig. 8), reflecting variations in magmatic source and tectonic environment. Samples such as Gh4A and Gh6, are characterized by relatively low Nb and Y values, plot near the boundary between within-plate granite (WPG) and volcanic arc granite (VAG) fields, suggesting transitional or mixed tectonic signatures. In contrast, samples like Gs1C and Gh2A lie closer to the arc-related domain, indicating contributions from subduction-modified mantle sources. ElGara samples cluster near the interface between arc-related and within-plate fields, consistent with a hybrid tectono-magmatic regime involving both crustal and mantle components (Fig. 9). The trace element and REE systematics of the ElGara granitoids point to magmas derived from crustally influenced, LREE-enriched sources emplaced within a transitional tectonic regime. The coexistence of arc-related geochemical signatures with within-plate characteristics (Fig. 15) implies evolution during the late stages of subduction or the onset of post-collisional extension. This dual affinity likely reflects progressive magmatic evolution during a shift from subduction-related to post-collisional extensional conditions. The integrated geochemical data therefore support derivation of the ElGara granitoids from evolved, crustally contaminated magmas that underwent fractional crystallization and assimilation during emplacement in a late- to post-orogenic extensional setting. These tectono-magmatic processes have direct implications for the geothermal potential of the ElGara plutons. The crustal contribution and strong magmatic differentiation promoted the concentration of heat-producing elements such as U, Th, and K, resulting in high radiogenic heat production (RHP) values that locally exceed **93.99 µW/m**^3^. The spatial association of these high-RHP zones with Th-enriched peraluminous facies (e.g., Gh6 and Gh4A) underscores the link between geochemical evolution and thermal characteristics. Consequently, the ElGara granites represent a promising geothermal resource within Egypt’s Southwestern Desert, capable of sustaining low-enthalpy energy systems and enhanced hydrothermal circulation within the upper crust.

## Role of microorganisms in radiation mitigation and environmental management

The elevated concentrations of naturally occurring radionuclides (^232^Th, ^238^U, ^40^K) in the ElGara granitoids necessitate integrated strategies for environmental radiation management. In addition to geological and engineering approaches, recent advances in geomicrobiology highlight promising biotechnological solutions. Extremophilic microorganisms such as *Deinococcus radiodurans* and *Rubrobacter radiotolerans* exhibit remarkable resistance to ionizing radiation and can survive doses far exceeding human tolerance^53–56^. These organisms can bind, immobilize, or transform radionuclides—including U, Th, and Cs—thereby reducing their mobility and environmental bioavailability through processes such as: biosorption; biomineralization; redox transformation and bioaccumulation. Their resilience arises from metabolic and structural adaptations, including: strong antioxidant systems; highly efficient DNA repair pathways (e.g., catalase, superoxide dismutase) and carotenoid and melanin pigments that provide radioprotective shielding^23^. Although these mechanisms cannot remediate radiation damage in human tissues, they provide blueprints for (1) bio-inspired radioprotective materials; (2) microbial bioremediation of radioactive soils; (3) biobarrier systems for radionuclide containment and (4) radiation-resistant biomaterials for occupational safety^26,57–59^.

Integrating geomicrobiological tools with geochemical monitoring could enhance the sustainable management of naturally radioactive terrains like ElGara. These systems complement conventional remediation techniques by stabilizing radionuclides, minimizing ecological exposure, and improving long-term environmental resilience. Thus, radiation-resistant microorganisms represent a promising frontier for mitigating natural radioactivity and enhancing environmental stewardship in granitic regions with elevated radiological signatures^23,37,58^.

## Conclusions

The El Gara granitoids of the Southwestern Desert exhibit the geochemical, mineralogical, and radiological characteristics of A-type, Fe-enriched tholeiitic granitoids emplaced during a post-orogenic extensional phase of the Arabian–Nubian Shield. Their compositional variability—from syenite and quartz-monzonite to syenogranite—reflects a complex magmatic system derived from both reduced, high-temperature crustal melts and mantle-influenced alkaline magmas, with subsequent differentiation and limited interaction between magmatic batches. The coexistence of peraluminous and peralkaline varieties is best explained by contributions from heterogeneous source regions combined with fractional crystallization and minor assimilation.

Activity concentrations of ^226^Ra, ^232^Th, and ^40^K are elevated and fall within the upper range of global A-type granite suites. These enrichments are consistent with accessory mineral assemblage characteristic of evolved anorogenic granitoids. Variability in radiogenic heat production (RHP), ranging from ~ 3 to 10 µW/m^3^, reflects the spatial distribution of heat-producing elements (U, Th, K) and confirms the significance of these plutons as local thermal contributors. The highest RHP values occur in the more evolved peralkaline facies, highlighting the strong link between magmatic differentiation, HFSE enrichment, and thermal potential.

Radiological indices such as Ra_eq_, D_γ_, H_in_, and H_ex_ exceed global reference values for rock units; however, these results represent rock-level radioactivity only. Actual public exposure depends on specific usage scenarios, material processing, shielding, ventilation, and occupancy patterns. Therefore, while the granitoids warrant caution for use in indoor construction, the environmental risk is potential rather than immediate, and site-specific assessments would be necessary before large-scale utilization.

The conceptual discussion of radiation-resistant microorganisms—particularly *Deinococcus radiodurans*—highlights emerging opportunities in biomineralization and biobarrier research, especially in radionuclide-rich geological settings. This perspective does not imply local biological activity but provides a scientifically grounded framework for future investigations into geomicrobiological responses to natural radiation fields.

Overall, this study provides the first integrated evaluation of petrogenesis, radiogenic heat production, and radiological characteristics of the El Gara plutons. These results contribute to regional understanding of A-type magmatism in the Arabian–Nubian Shield while offering important implications for geothermal assessment, environmental radiation management, and future research on microbe–radiation interactions.

### Limitations and future work

Although this study provides the first integrated assessment of the petrogenesis, radiogenic heat production, and radiological characteristics of the El Gara granitoids, several limitations must be acknowledged. First, the gamma-spectrometric analysis was conducted on a limited number of representative samples, while these capture the full geochemical range of the plutons, a denser sampling grid would allow finer spatial resolution of radioelement distribution and RHP variability. Second, the study relies on the assumption of secular equilibrium within the U- and Th-decay chains; although samples were sealed for 28 days, deviations from equilibrium in natural systems may introduce minor uncertainties in activity calculations. Third, no mineral-specific U–Th–K analyses (e.g., via LA-ICP-MS or EPMA) were performed, which limits the ability to fully quantify the contributions of zircon, allanite, monazite, and other accessory minerals to total heat production.

Furthermore, the discussion of microbial radiation tolerance is conceptual and not supported by local microbiological data. In situ biological sampling, metagenomic analysis, and culture-based assays would be required to validate ecological responses to the measured radiation fields. Similarly, the environmental dose implications presented here are based on rock-level activities rather than field measurements of radon exhalation, gamma dose rates in air, or realistic indoor exposure scenarios. Future work should, therefore, focus on (1) expanding the geochemical and radiological dataset through high-resolution sampling across both plutons; (2) applying mineral-scale analytical techniques to establish precise U–Th–K hosts and improve thermal modeling; (3) performing in situ environmental radiation mapping and radon flux measurements; (4) evaluating indoor and outdoor exposure scenarios for potential building-material use; and (5) conducting geomicrobiological investigations to assess whether radiation-resistant microbial communities are present in or around the El Gara granitoids. Such integrated studies would greatly enhance understanding of the geological evolution, geothermal significance, and environmental behavior of these A-type granitoids.

## Data Availability

All datasets generated or analyzed during the current study are included in this published article. Additional raw gamma-spectrometric data and geochemical spreadsheets are available from the corresponding author upon reasonable request.
